# Seipin deficiency-induced lipid dysregulation leads to hypomyelination-associated cognitive deficits via compromising oligodendrocyte precursor cell differentiation

**DOI:** 10.1038/s41419-024-06737-z

**Published:** 2024-05-21

**Authors:** Wenli Cui, Jing Yang, Chuanyun Tu, Ziting Zhang, Huifang Zhao, Yan Qiao, Yanqiu Li, Wulin Yang, Kah-Leong Lim, Quanhong Ma, Chengwu Zhang, Li Lu

**Affiliations:** 1 School of Basic Medical Sciences, Shanxi Medical University, Taiyuan, 030001 Shanxi China; 2 Key Laboratory of Cellular Physiology, Ministry of Education, Shanxi Medical University, Taiyuan, 030001 Shanxi China; 3 Analytical Instrumentation Center & State Key Laboratory of Coal Conversion, Institute of Coal Chemistry, Chinese Academy of Sciences, Taiyuan, 030001 Shanxi China; 4 Center of Medical Physics and Technology, Hefei Institutes of Physical Science, Chinese Academy of Sciences, Hefei, 230031 China; 5 Lee Kong Chian School of Medicine, Nanyang Technological University, 11 Mandalay Road, Singapore, 308232 Singapore; 6 Jiangsu Key Laboratory of Neuropsychiatric Diseases, Institute of Neuroscience, Soochow University, Suzhou, Jiangsu China

**Keywords:** Oligodendrocyte, Myelin biology and repair

## Abstract

Seipin is one key mediator of lipid metabolism that is highly expressed in adipose tissues as well as in the brain. Lack of Seipin gene, *Bscl2*, leads to not only severe lipid metabolic disorders but also cognitive impairments and motor disabilities. Myelin, composed mainly of lipids, facilitates nerve transmission and is important for motor coordination and learning. Whether Seipin deficiency-leaded defects in learning and motor coordination is underlined by lipid dysregulation and its consequent myelin abnormalities remains to be elucidated. In the present study, we verified the expression of Seipin in oligodendrocytes (OLs) and their precursors, oligodendrocyte precursor cells (OPCs), and demonstrated that Seipin deficiency compromised OPC differentiation, which led to decreased OL numbers, myelin protein, myelinated fiber proportion and thickness of myelin. Deficiency of Seipin resulted in impaired spatial cognition and motor coordination in mice. Mechanistically, Seipin deficiency suppressed sphingolipid metabolism-related genes in OPCs and caused morphological abnormalities in lipid droplets (LDs), which markedly impeded OPC differentiation. Importantly, rosiglitazone, one agonist of PPAR-gamma, substantially restored phenotypes resulting from Seipin deficiency, such as aberrant LDs, reduced sphingolipids, obstructed OPC differentiation, and neurobehavioral defects. Collectively, the present study elucidated how Seipin deficiency-induced lipid dysregulation leads to neurobehavioral deficits via impairing myelination, which may pave the way for developing novel intervention strategy for treating metabolism-involved neurological disorders.

## Introduction

Seipin, a conserved endoplasmic reticulum transmembrane protein, is highly expressed in adipose tissues and the brain. Seipin affects cellular lipid homeostasis directly by regulating the enzyme machinery of fatty acid (FA) synthesis, phosphatidic acid (PA) metabolism, or sphingolipid production [1–4], mediating phosphatidylcholine (PC) synthesis [5], or regulating lipolysis [6]. Lipid droplets (LDs), crucial dynamic organelles for lipid homeostasis. Across various species, ranging from yeast to humans, Seipin plays evolutionary conserved roles in LDs biogenesis and degradation [7–9]. Mutation or deficiency Seipin result in lipid dyshomeostasis, causing accumulation of PA, PC, ceramide (Cer), triglyceride (TAG), and formation of aberrant LDs [5, 10, 11]. Nonsense mutations in *Bscl2*, the gene encoding Seipin, lead to a severe Congenital Generalized Lipodystrophy type 2 (CGL2) disease, clinically manifested with lipodystrophy, mental retardation and cognitive decline [12]. These clinical symptoms are phenocopied by Seipin-deficient rodents, displaying systemic metabolic disorders [13, 14] and neurological deficits, including spatial cognition decline and motor coordination deficits. Underlying mechanisms involve suppressed synaptic transmission, adult neurogenesis inhibition, and overreactive neuroinflammation [15–19]. Aside from these observations, patients carrying Seipin mutations develop multiple demyelinating symptoms, including a reduced number of large myelinated fibers, thinning of myelin sheaths, nerve conduction blockage, and reduced conduction velocity [20]. These symptoms closely correlate with the integrity and quantity of myelin, a membranous structure composed of 70–85% lipids, primarily sphingolipids and cholesterol [21].

In the brain, myelin formed by oligodendrocytes (OLs) predominantly occurs postnatally, with ongoing generation of newborn OLs to sustain adaptive myelination. Neuronal activity could promote oligodendrogenesis [22], facilitating rapid myelination during learning and memory [23, 24]. In addition, preexisting OLs contribute to cognitive maintenance by altering the pattern of axonal myelination, termed myelin plasticity [25]. However, the mechanisms underlying the regulation of myelin plasticity remain unclear. Lipid homeostasis is crucial for not only proper myelination [26, 27], also OL precursor cell (OPC) differentiation [28, 29]. Abnormal lipid metabolism leads to OLs defects and myelin abnormalities [30]. Knockout of lipogenic enzymes gene (e.g., *Fa2h*, *Fasn*) in mice disrupts OL differentiation and causes late-onset myelin splitting or demyelination [31, 32]. Moreover, in Alzheimer’s disease, and Parkinson’s disease, Seipin expression decreased, accompanied by lipid dysregulation and myelin deficits [33–36], All those evidences suggest Seipin, as a vital lipid metabolism mediator, play essential role in myelin plasticity. However, whether and how Seipin deficiency-leaded neurobehavioral impairments is underlined by lipid dysregulation and its consequent myelin abnormalities remains to be elucidated.

Here we presented compelling evidence showing that Seipin deficiency in mice resulted in impaired spatial cognition & motor coordination, myelin abnormalities, and impeded OPC differentiation. Genetically reducing Seipin expression in OLN cells (rat OPCs line) down-regulated lipid metabolism-related genes, disrupted intracellular LDs dynamics, and impaired OLN cell differentiation. Importantly, pharmacological activation of lipid metabolism via rosiglitazone (RG) successfully restored phenotypes associated with Seipin deficiency, including restoring LDs dynamics, replenishing sphingolipids, prompting OPC differentiation, and ameliorating neurobehavioral defects. Taken together, our findings provide insights into the intricate relationship between Seipin deficiency-associated lipid dysmetabolism and myelin defects, offering novel clues for potential intervention of Seipin deficiency-related neurological disorders.

## Results

### Seipin deficiency resulted in deficits in cognition and motor coordination of mouse

**Fig. 1:**
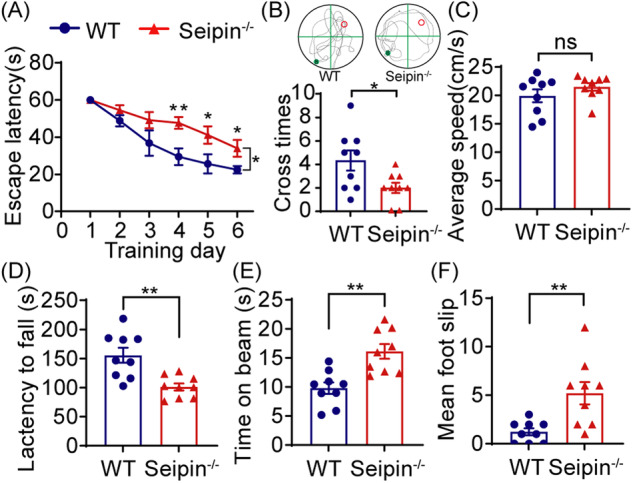
Seipin deficiency impaired cognition and motor coordination of mice. **A-C** The Morris water maze test shows the latency to reach the hidden platform in the acquisition phase (**A**), the number of platform crossings in the target quadrant and representative tracks (**B**), and average swimming speed (**C**) in the Seipin^-/-^ and WT mice. **D** Latency to fall off in the rotarod test. **E**, **F** Time to cross the beam (**E**) and number of foot slips (**F**) in the beam walking test. Graph data were presented as mean ± s.e.m. with n = 9 mice/group. *P < 0.05, **P < 0.01 **(A** Two-way repeated ANOVA was used for the latencies to platform from the 1^st^ to 6^th^ days. **A-F** Unpaired *t*-tests were used for *t*he outcomes on the 4^th^, 5^th^, and 6^th^ day).

Patients with Seipin gene mutation exhibit not only metabolic dyshomeostasis but also neurobehavioral impairments [12]. To assess the impacts of Seipin deficiency on rodent cognitive function and motor coordination, Seipin^-/-^ mice were created and subjected to a series of behavioral tests. In the MWM test, Seipin^-/-^ mice exhibited longer escape latency to find the hidden platform, and spent less time in the spot where the platform was located compared to WT mice, indicating impaired spatial learning and memory (Fig. 1A, B). Swimming speed was comparable between Seipin^-/-^ mice with WT mice (Fig. 1C), excluding the possibility that above phenotypes were due to the difference in swimming speed. Moreover, Seipin^-/-^ mice showed lower spontaneous alternation behavior in Y-maze (Fig. S1A-C), and less preference for the novel object in novel object recognition test, indicating impaired recognition memory (Fig. S1D-F). We then measured their motor performance. In the rotarod assay, Seipin^-/-^ mice showed shorter latency of falling from rotarod than that of WT mice (Fig. 1D). In the beam walking test, Seipin^-/-^ mice displayed increased foot slips and longer latency to transverse narrow beam (Fig. 1E, F). Above results suggested that Seipin deficiency impaired the cognitive function and motor coordination of mice.

### Seipin deficiency resulted in hypomyelination in the brain

**Fig. 2:**
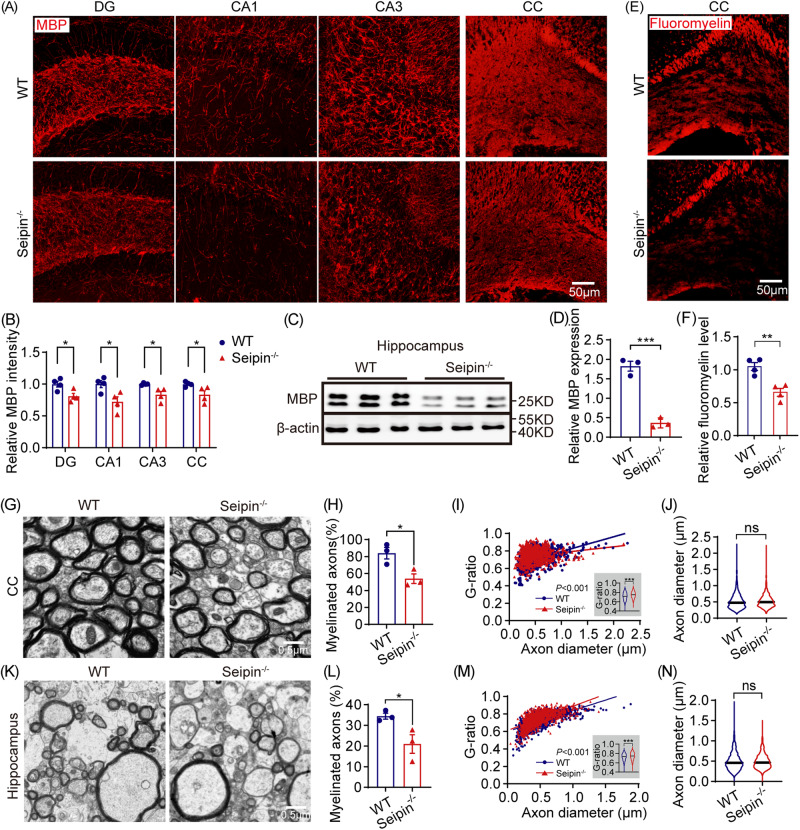
Seipin deficiency induced hypomyelination in mice brain. **A, B** Representative images (**A**) and quantification of MBP signal intensity (**B**) in the DG/CA1/CA3 of the hippocampus and CC of Seipin^-/-^ and WT mice. **C, D** Immunoblot blots (**C**) and densitometric analysis (**D**) of MBP in hippocampus of Seipin^-/-^ and WT mice. **E, F** Representative images (**E**) and quantification (**F**) of Fluoromyelin signal intensity in the CC of Seipin^-/-^ and WT mice. **G-J** Representative electron micrographs (**G**), proportion of myelinated axons (**H**), scatter plots of g-ratio values across all axon diameters (**I**), and axon diameters (**J**) in the CC of Seipin^-/-^ and WT mice. **K-N** Representative electron micrographs (**K**), quantification of the percentage of myelinated axons (**L**), scatter plots of g-ratio across all axon diameters (**M**), and axon diameters (**N**) in the hippocampus of Seipin^-/-^ and WT mice. Graph data were presented as mean ± s.e.m. with n = 3-4 mice per group (**B-F**) and n > 850 axons from 3 mice/group (**G-N**). *P < 0.05, **P < 0.01; ***P < 0.001 (Unpaired *t*-tests).

Given the above neurobehavioral deficits in Seipin^-/-^mice and myelin’s crucial role in maintaining brain function, we assessed myelin morphology under Seipin deficiency. As shown in Fig. 2A–D, immunofluorescence and western blotting revealed substantial reduction of MBP, a myelin hallmark protein, in the hippocampus of Seipin^-/-^ mice. Fluoromyelin staining indicated significantly weaker signals in the hippocampus and CC of Seipin^-/-^ mice, suggesting reduced myelin lipids (Fig. 2E, F). TEM analysis showed a decreased proportion of myelinated axons in the CC of Seipin^-/-^ mice, along with thinner myelin sheaths and the comparable axon diameter (Fig. 2G–J). Hippocampal myelin sheaths in Seipin^-/-^ mice also exhibited structural abnormalities (Fig. 2K–N). These results indicated that Seipin deficiency led to brain hypomyelination.

### Seipin deficiency caused reduced OL density in mice brain

**Fig. 3:**
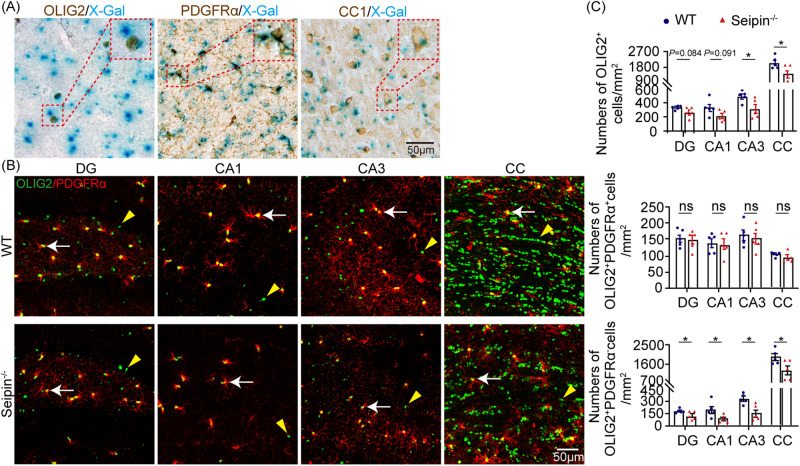
Seipin deficiency reduced OL density in mice brains. **A** Representative images of OLIG2, PDGFRα, or CC1 staining (brown) and X-gal staining (blue) of Seipin^-/-^ mice brain sections. Red dotted boxes show the enlarged fields. **B** Representative merged images of OLIG2 (green) and PDGFRα (red) double staining. OPCs (OLIG2^+^PDGFRα^+^) are indicated by white arrows. OLs (OLIG2^+^PDGFRα^-^) are indicated by yellow arrowheads. **C** Quantification of OL lineage cells (OLIG2^+^), OPCs (OLIG2^+^PDGFRα^+^), and OLs (OLIG2^+^PDGFRα^-^) per mm^2^ in the DG, CA1, CA3, and CC. Graph data were presented as mean ± s.e.m. with n = 5 mice/group. *P < 0.05; ns, not significant (Unpaired *t*-tests).

To explore the mechanism underlying hypomyelination in the brain of Seipin^-/-^ mice, we investigated Seipin expression in OL lineage cells using X-gal staining with a lacZ cassette. LacZ-positive immunoreactivity was evident in OLIG2-positive OL lineage cells, PDGFRα-positive OPCs, and CC1-positive OLs (Fig. 3A). Also, *Bscl2* mRNA expression, examined by RNAScope, aligned with OL lineage cells (Fig. S2A-C). To examine the potential role of Seipin in OL lineage cells, we performed immunofluorescence staining to analyze density of these cells. The results revealed decreased densities of OLIG2^+^ OL lineage cells and OLIG2^+^PDGFRα^-^ OLs in hippocampal subareas and CC of Seipin^-/-^ mice compared to WT mice. However, OLIG2^+^PDGFRα^+^ OPC density exhibited comparable (Fig. 3B, C). These data indicated that Seipin deficiency reduced OL density, which compromised myelination.

### Seipin deficiency impaired OPC differentiation

**Fig. 4:**
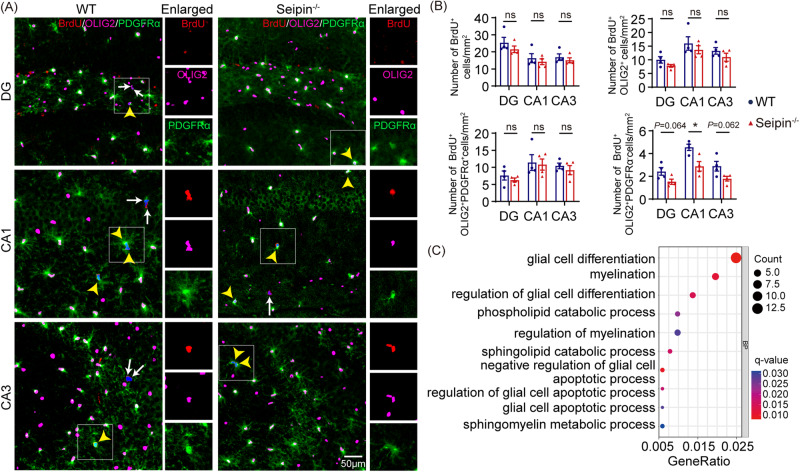
Seipin deficiency compromised OPC differentiation in mice brains. **A** Representative images illustrating triple-staining of BrdU (red) with OL linage marker OLIG2 (pink) and OPCs marker PDGFRα (green). Yellow arrowheads, proliferating OPCs (BrdU^+^OLIG2^+^PDGFRα^+^). White arrows, newly generated OLs (BrdU^+^OLIG2^+^PDGFRα^-^). **B** Quantification of proliferating cells (BrdU^+^), proliferating OL linage cells (BrdU^+^OLIG2^+^), proliferating OPCs, and newly generated OLs per mm^2^ in the DG, CA1, and CA3. **C** For GO functional analysis of all DEGs in isolated brain tissues from Seipin^-/-^ relative to WT mice, scatter plots show items significantly enriched in the biological process (BP). Graph data were presented as mean ± s.e.m. with *n* = 4 mice/group. *P < 0.05; ns, not significant (Unpaired *t*-tests).

Given that the differentiation of OPCs dominates the number of OLs and myelination, we explored whether reduced OL density in the brains of Seipin^-/-^ mice was due to impairment of OPC differentiation. For this purpose, mice were intraperitoneally injected with BrdU for 7 days and sacrificed 14 days’ post-injection. The density of BrdU^+^ cells, BrdU^+^OLIG2^+^ cells, and BrdU^+^OLIG2^+^PDGFRα^+^ cells in the hippocampus of Seipin^-/-^ mice showed no difference from those in WT mice, indicating that Seipin deficiency did not affect the proliferation of OPCs. However, a significant reduction in BrdU^+^OLIG2^+^PDGFRα^-^ newborn OL density occurred in Seipin^-/-^ hippocampus CA1, with a similar trend in DG and CA3 (Fig. 4A, B). GO enrichment analysis of the transcriptome from the brains of adult Seipin^-/-^ and WT mice also showed that several DEGs were enriched in myelination-related processes, such as glial cell differentiation, myelination, regulation of myelination, and sphingolipid metabolic process (Fig. 4C). These results suggested crucial role of Seipin in maintaining OPC differentiation, but not proliferation.

To ascertain the role of Seipin in OPC differentiation, we used the in vitro OPCs cell line—OLN cells model. As shown in Fig. S3A-G, upon the conditional medium (EBSS), OLN cells differentiated into multipolar processes, and Seipin mRNA and protein expression increased in differentiated OLN cells along with upregulated MAG, a featured protein of mature OLs, revealing the potential regulatory role of Seipin in OLN cell differentiation. To validate Seipin’s involvement, OLN cells were subjected to lentiviral Seipin-shRNA treatment. Seipin-shRNA-treated cells showed significantly reduced Seipin mRNA and protein compared to NC-shRNA-treated cells (NC) (Fig. S4A-D). After inducing differentiation for 24 h, Seipin-shRNA-treated OLN cells exhibited lower proportions of well-differentiated cells with extensive branches and networks (G3) (10% vs. 45%), while the poorly differentiated cells with only two primary processes (G1) increased (3% vs. 49%) compared to the control group (Fig. S4E-G). Additionally, mRNA levels of *Mag* and *Plp1*, featured genes of mature OLs, were reduced in the Seipin-shRNA treated cells compared to the control (Fig. S4H). These results further demonstrated that Seipin deficiency impaired OPC differentiation.

### Intracellular LDs dynamics and sphingolipid metabolism modulated OPC differentiation

**Fig. 5:**
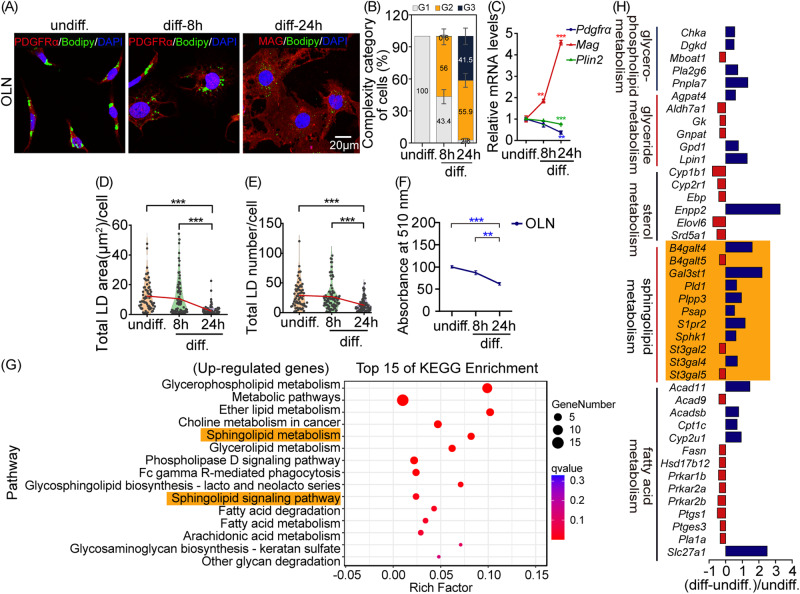
LDs and sphingolipid metabolism were involved in OPC differentiation. **A** Representative fluorescent images show LDs in OLN cells with differentiation for 0, 8, or 24 h. **B** Percentage histogram of different histological categories of OLN cells. **C** Normalized levels of *Pdgfrα*, *Mag*, and *Plin2* mRNA. asterisk, diff-24 versus undiff. or diff-8h versus undiff. **D, E** Violin plots depict area (**D**) and number (**E**) of LDs per cell. The mean values of LDs area and number at different times are connected by a red line. **F** Quantification of ORO staining. **G** Scattered plots show the top 15 pathways significantly enriched for all DEGs between diff- and undiff-OLN cells. **H** RNA-seq shows the fold changes of significantly altered sphingolipid metabolism-related genes (orange patch) in OLN cells after 24 h of differentiation. undiff.: undifferentiated. diff.: differentiated for 24 h. Graph data were presented as mean ± s.e.m. n > 150 cells (**B, D, and E**). **P < 0.01, ***P < 0.001 (One-way ANOVA and followed by Bonferroni’s post hoc test).

LDs, organelles that reflects the homeostasis of cellular lipid metabolism, were present in OL lineage cells, but its function had not been robustly explored [37–39]. To reveal the potential role of LDs in regulating OPC differentiation, we examined LDs morphology and content in OLN cells at two distinct time points-8 h and 24 h post-induction of differentiation. Quantitative analysis of cell morphology revealed a transition in cell morphology from initial bifurcated branching (G1) to secondary branching (G2), and later to multiple branching or a mesh-like appearance (G3), indicating continuous OLN cell maturation during prolonged differentiation (Fig. 5A, B). Expression of Plin2, a specific LDs coat protein, decreased in differentiating OLN cells over time (Fig. 5C). Co-staining with Bodipy-493/503 showed a time-dependent reduction in LDs area and number in differentiating OLN cells, compared to undifferentiated ones (Fig. 5D, E). Simultaneously, ORO staining for neutral lipids confirmed a time-dependent decrease in content within differentiating OLN cells (Fig. 5F). Collectively, these findings suggested LDs exhibited time-dependent reduction in OLN cell differentiation.

We next tested whether artificially increasing LDs or inhibiting lipolysis could alter OLN cell differentiation. Exogenous fatty acids, such as Oleic acid (OA), can be uptaken by cells and induce LDs formation. We added OA to OLN cell cultures and its impact was assessed. It led to a 1-fold increase in LDs area, evidenced by Bodipy-positive signals (Fig. S5A-C). This led to an augmentation of medium and larger-sized LDs (Fig. S5D), demonstrating that LDs content in OLN cells could be artificially altered. Upon inducing differentiation for 24 h, LDs areas significantly decreased, accompanied by a reduction in the proportion of larger-sized LDs (Fig. S5A-D), suggesting the consumption of larger-sized LDs during differentiation. Notably, the proportion of differentiated cells in G2 and G3 states increased in OA-treated OLN cells (Fig. S5E). These results collectively indicated that the increasing lipid load in OPCs contributed to its differentiation.

To further confirm the impact of LDs on OLN cell differentiation, we utilized Atglistatin, a lipolysis inhibitor to block LDs degradation. Atglistatin significantly increased LDs accumulation in OA-treated OLN cells compared to the control (Fig. S5F, G). Furthermore, the proportion of highly differentiated cells was significantly reduced by Atglistatin treatment, indicating the crucial role of LDs utilization in OPC differentiation (Fig. S5H). These results highlighted the importance of both LDs content and LDs utilization in OPC differentiation.

To uncover the genes involved in lipid metabolism in OLN cell differentiation, transcriptome analysis was performed. KEGG analysis revealed that up-regulated genes enrichment in lipid metabolism pathways, particularly sphingolipid metabolism (Fig. 5G). Notably, genes related to sphingolipid metabolism, such as *B4galt4*, *Plpp3*, *Sphk1*, *S1pr2*, and *St3gal4*, were significantly upregulated during OLN cell differentiation (Fig. 5H). These results suggested that sphingolipid metabolism played an important role in OPC differentiation.

### Seipin deficiency compromised LDs utilization & sphingolipid metabolism in OPC differentiation in vitro and in vivo

**Fig. 6:**
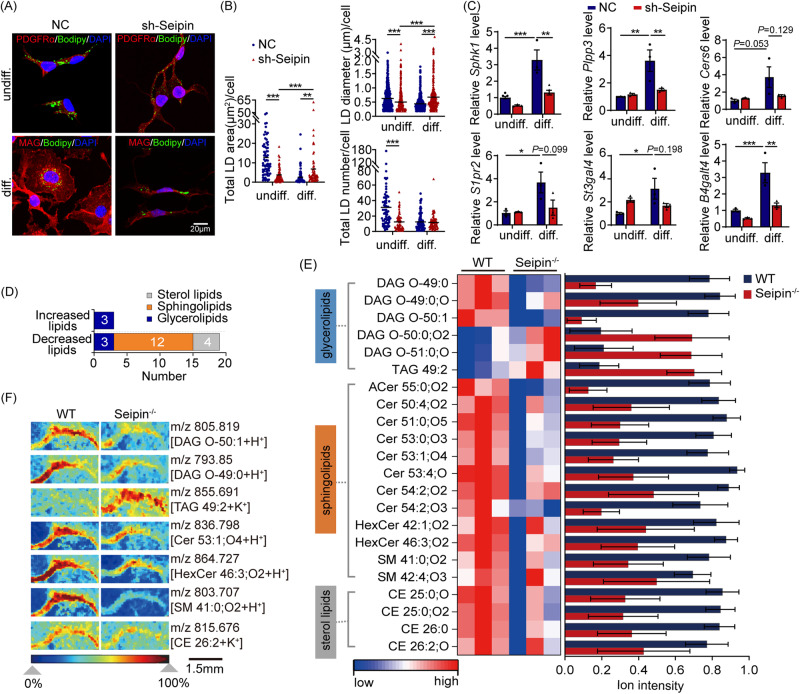
Seipin deficiency compromised LDs dynamics & sphingolipid metabolism in OPC differentiation. **A** Representative confocal images of NC and sh-Seipin OLN cells before and after differentiation. **B** Violin plots illustrate area, diameter, and number of LDs per cell in NC and sh-Seipin OLN cells before and after differentiation. Black lines, mean values of LDs area, diameter, or number. **C** Normalized mRNA levels of sphingolipid metabolism-related genes in NC and sh-Seipin OLN cells before and after differentiation. **D–F** MALDI-TOF MSI shows distribution of identified lipids (**D**), heatmap analysis of significantly changed lipids (**E**), and ion images of representative lipids (**F**) in the CC of adult Seipin^-/-^ mice. undiff.: undifferentiated. diff.: differentiated for 24 h. Graph data were presented as mean ± s.e.m. with *n* = 3 mice/group (**D–F**). *P < 0.05, **P < 0.01, ***P < 0.001 (**B, C** Two-way ANOVA followed by Turkey-Kramer post hoc tests).

Seipin affects intracellular LDs homeostasis by regulating the metabolism of sphingolipids and other lipids [1, 5, 10]. To investigate whether the impaired differentiation of OPC due to Seipin deficiency is associated with alterations in intracellular LDs dynamics and sphingolipid metabolism, we evaluated LDs features and sphingolipid metabolism-related gene expression in OLN cells of Seipin knockdown. We found that Seipin knockdown reduced LDs area in OLN cells compared to the control (Fig. 6A, B). In differentiated sh-Seipin cells, LDs area and size significantly increased, while LDs number remained unchanged (Fig. 6B). Notably, Seipin knockdown down-regulated expression of sphingolipid metabolism-related genes (*Sphk1*, *Plpp3*, *Cers6*, *S1pr2*, *St3gal4*, and *B4galt4*) in OLN cells (Fig. 6C). These results suggested that Seipin deficiency disrupted sphingolipid metabolism and LDs utilization, impeding OLN cell differentiation.

Triggered by the in vitro observations, we analyzed the transcriptomics and lipidomics of the Seipin^-/-^ mice brain. The transcriptome analysis showed that genes related to lipid synthesis and transport (*Abca1, Abca4, Abca8a, Fabp7, Creb1*), ceramide and glycosphingolipid synthesis (*Cers6, B3gnt2, B4galt4*), sphingolipid metabolism (*Map3k1*, *Sphk1*) were down-regulated (Fig. S6A, B). Lipidomic profile was obtained via MALDI-TOF MSI method in positive-ion mode, which is a well-accepted imaging technique for visualizing the spatial distribution of endogenous lipid molecules [40]. As shown in Fig. 6D, 22 species of lipids were found to be significantly altered in CC of adult Seipin^-/-^ mice, of which sphingolipids accounted for more than half of them. Specifically, 3 glycerides, 12 sphingolipids and 4 sterols were down-regulated, and 3 glycerides were up-regulated (Fig. 6E). MSI based on the total ion current vividly showed the decrease of sphingolipids including Cer (53:1;O4), SM (41:0;O2) and HexCer (46:3;O2) (Fig. 6F). These results suggested that Seipin deficiency compromised sphingolipid metabolism in vitro and in vivo.

### RG rescued Seipin deficiency-induced phenotypes in vitro and in vivo

**Fig. 7:**
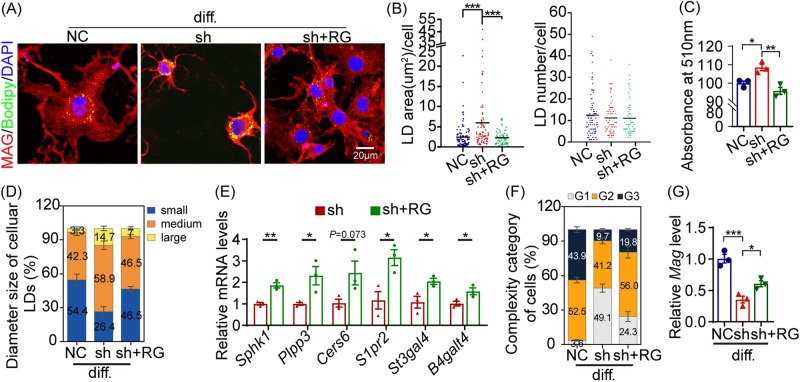
RG treatment promoted OPC differentiation by restoring LDs dynamics and sphingolipid metabolism in OLN cells. **A** Representative images of differentiated NC, sh-Seipin (sh), and sh+RG cells. **B** Violin plots illustrate the area and number of LDs in differentiated NC, sh, and sh+RG groups. Black lines, mean values of LDs area and number. **C** Quantification of ORO staining. **D** Distribution of different-sized LDs in differentiated NC, sh, and sh+RG cells. **E** Normalized mRNA levels of sphingolipid metabolism-related genes in differentiated sh and sh+RG cells. **F** Percentage histogram of histological categories of differentiated NC, sh, and sh+RG cells. **G** Normalized *Mag* mRNA levels in differentiated NC, sh, and sh+RG cells. diff.: differentiated for 24 h. NC: OLN cells transfected with NC-shRNA. sh: OLN cells transfected with Seipin-shRNA. sh+RG: sh-Seipin cells treated with RG. Graph data were presented as mean ± s.e.m. n > 150 cells (**B, D, and F**). *P < 0.05, **P < 0.01, ***P < 0.001 (**B, C, and G** One-way ANOVA followed by Bonferroni’s post hoc test. **E** unpaired *t*-test).

After observing the deficits induced by Seipin deficiency in vitro and in vivo, we were eager to explore how to rescue those phenotypes. RG, a classic PPARγ activator, was reported to regulate lipid metabolism [41]. Here, we administered RG and assessed its potential effects on lipid metabolism as well as OPC differentiation, and neurobehavioral manifestation in vitro and in vivo. As shown in Fig. 7A, B, RG treatment resulted in a significant reduction in LDs accumulation in differentiated sh-Seipin cells, with no obvious effect on LDs number. Quantification of ORO stain confirmed RG treatment decreased neutral lipid levels in sh-Seipin cells (Fig. 7C). RG treatment successfully restored the proportion of LDs in different size in differentiated sh-Seipin cells (Fig. 7D), implying enhanced utilization of these LDs. Meanwhile, RG treatment up-regulated expression of sphingolipid metabolism-involved genes (*Sphk1*, *Plpp3*, *CerS6*, *S1pr2*, *St3gal4*, and *B4galt4*) in differentiated sh-Seipin cells (Fig. 7E). Additionally, RG treatment induced an elevation in the proportion of G2 and G3 state differentiated cells (Fig. 7F), and up-regulated *Mag* mRNA expression in sh-Seipin cells (Fig. 7G), indicating that RG treatment effectively accelerate OLN cell differentiation compromised by Seipin-knockdown. These data demonstrated that RG administration effectively promoted OPC differentiation in the content of Seipin deficiency by regulating sphingolipid metabolism and LDs utilization.

**Fig. 8:**
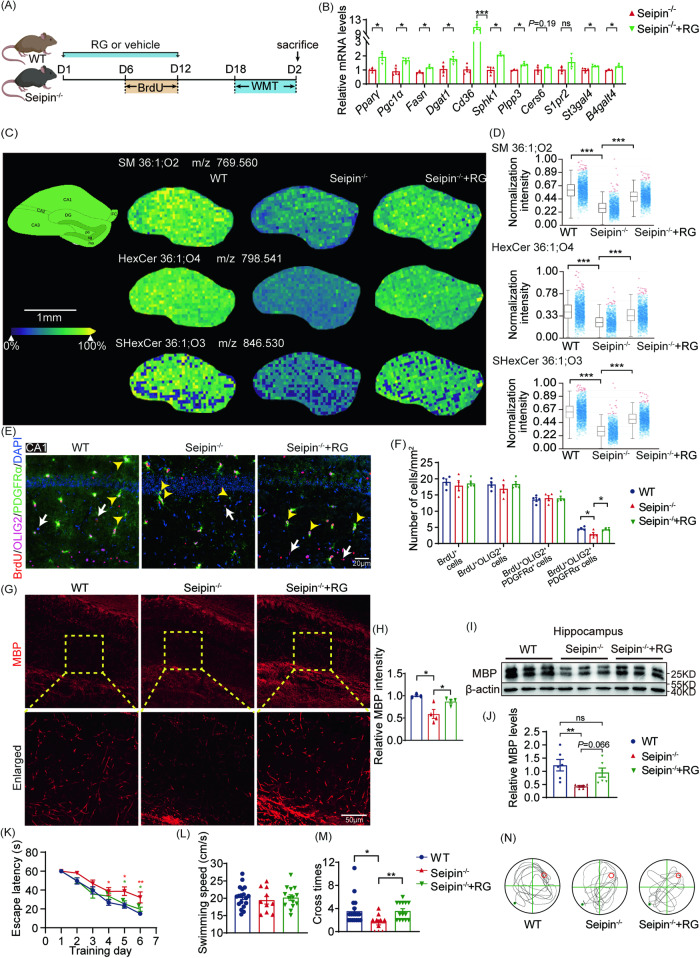
RG treatment improved myelination and cognition by restoring sphingolipid metabolism of Seipin^-/-^ mice. **A** Illustration of experimental schedule. **B** Relative mRNA levels of sphingolipid metabolism-related genes in Seipin^-/-^ mice upon treatment with RG (Seipin^-/-^+RG). **C, D** Spatial distribution (**C**) and box plots (**D**) show ions intensity of sphingolipids in the hippocampus. AUC > 0.75, ***P < 0.001. **E, F** Representative images (**E**) and quantification (**F**) show the total number of proliferating OL linage cells (BrdU^+^OLIG2^+^), proliferating OPCs (BrdU^+^OLIG2^+^PDGFRα^+^), and newly generated OLs (BrdU^+^OLIG2^+^PDGFRα^-^) in the CA1. **G, H** MBP staining (**G**) and quantification (**H**) of CA1. Dashed boxed areas are enlarged. **I, J** Western blotting (**I**) and quantification (**J**) of the bands of MBP in the hippocampus. **K** Latency to reach the hidden platform in the MWM test. red asterisk, WT versus Seipin^-/-^ mice; green asterisk, Seipin^-/-^ versus Seipin^-/-^+RG mice. **L-N** Average swimming speed (**L**), the number of platform crossings in the target quadrant (**M**), and representative tracks (**N**) of WT, Seipin^-/-^, and Seipin^-/-^+RG mice in the MWM test. Graph data were presented as mean ± s.e.m. with n = 4-6 mice/group (**B-J**) and n = 10-18 mice per group (**K-N**). *P < 0.05, **P < 0.01, ***P < 0.001 (**B** Unpaired student’s *t*-test. **K** two-way repeated ANOVA followed by Bonferroni’s post hoc test. **D, F, H, J, L** and **M** one-way ANOVA followed by Bonferroni’s post hoc tests).

Based on promising in vitro results, we further investigated the effects of RG treatment on the neurobehavioral phenotypes of Seipin^-/-^ mice. As expected, RG treatment significantly upregulated PPAR*γ* downstream genes (*Pgc1α, Fasn, Dgat1 and Cd36*), and sphingolipid metabolism-associated genes (*Sphk1, Plpp3, Cers6, S1pr2, St3gal4, B4galt4*) in the hippocampus of Seipin^-/-^ mice compared to corn oil-treated control (Fig. 8A, B). MALDI-TOF MSI showed augmentation of sphingolipid lipids (SM, HexCer, and SHexCer) with RG treatment, which were down-regulated in the hippocampus of Seipin^-/-^ mice (Fig. 8C, D). Notably, newborn OLs (BrdU^+^OLIG2^+^PDGFRα^-^) in the hippocampal CA1 region of Seipin^-/-^+RG mice remarkably increased compared to untreated Seipin^-/-^ mice (Fig. 8E, F). RG treatment attenuated MBP loss in the hippocampus of Seipin^-/-^ mice (Fig. 8G–J). In the Morris water maze test, RG treatment significantly shortened the latent period of searching the hidden platform and increased the number of shuttles to the original platform during spatial exploration in Seipin^-/-^ mice (Fig. 8K–N). Moreover, there was significant increase in latency to fall off the rotarod, and notable reduction in foot slips and longer latency to transverse beam in the beam walking assay in Seipin^-/-^+RG mice compared to vehicle-treated Seipin^-/-^ mice, highlighting the rescue effects of RG treatment on motor coordination (Fig. S7A, B). These data demonstrated that RG treatment effectively rescued phenotypes associated with Seipin deficiency both in vitro and in vivo.

## Discussion

Seipin loss-of-function mutations cause severe metabolic disturbances and neurological abnormalities in humans [12]. Seipin knockout mice also exhibit neurological deficits [19, 42], emphasizing its crucial role in the nervous system. Our study revealed Seipin expression in OPCs and OLs in vitro and in vivo, aligning with single-cell RNA-seq data obtained from human, rodent tissues or primary rat OL lineage cells [43–45]. As OPCs and OLs underlie the myelin plasticity, crucial for cognition and motor coordination [46], we explored Seipin’s impact on this process. Our results demonstrated that Seipin deficiency compromised oligodendrogenesis and impaired myelin structure and function. Zhou et al. reported that Seipin deficiency impaired neuronal synaptic dysfunction and the NMDA receptor-dependent LTP in the hippocampus, resulting in spatial and cognitive deficits [19]. Ours and Zhou’s results all proved that Seipin deficiency impaired the cognitive function, but from different aspects, myelin plasticity and synaptic plasticity, respectively. More than that, it had been reported that OLs regulated glutamate vesicle release and LTP production at presynaptic terminals, and myelination could facilitate synaptogenesis, ultimately promoting in synaptic integration and remodeling of neuronal networks [47, 48]. Moreover, Seipin down-regulation was observed in aging and neurodegenerative diseases [33–35, 49, 50], and patients carrying Seipin mutations exhibited peripheral demyelinating symptoms [20]. These findings indicated that Seipin played important roles in brain plasticity and could serve as a potential target for intervention of neurological disorders.

LDs, crucial intracellular lipid reservoirs, maintain cellular lipid homeostasis by storing lipid precursors, regulating enzyme activity, providing substrates, and interacting with cell membranes [51, 52]. LDs were found in OL lineage cells, but their function had not been robustly explored [39, 53]. Our study confirmed the existence of LDs in OPCs and OLs, with more LDs in OPCs than premyelinating OLs. Moreover, exogenous lipid administration enhanced LDs synthesis, promoting OPC differentiation, while inhibiting LDs degradation hindered OPC differentiation. Additionally, in neural stem/progenitor cells, LDs dynamics were found to influence their proliferation [54]. Our results and others indicated that LDs dynamics played vital roles in regulating cellular behaviors.

Seipin was proved to get involved in LDs biogenesis, nucleation, transportation, and degradation [55–58]. Seipin deficiency inhibited LDs biogenesis during adipogenesis [9, 59] and impaired lipolysis in yeast [60] or mouse adipocytes [61]. In the present study, we found that Seipin deficiency impaired OLN cell differentiation. The content and size of LDs in undifferentiated OLN cells was significantly decreased in undifferentiated OLN cells, while increased in differentiated OLN cells. There were literatures reported that LDs size was influenced by lipid metabolism, of which Seipin was a key mediator [6, 62, 63]. These findings indicated that Seipin deficiency obstructed the degradation and utilization of LDs in OPCs, which compromised OPC differentiation.

Seipin modulates lipid metabolism, inhibiting glycolipid and PA formation in mammalian cells by binding and inhibiting GPAT3/4, the rate-limiting enzyme of glycolipid synthesis [64]. It also restricts PC synthesis by influencing the B12-one-carbon cycle-PC pathway in *C. elegans* strains [5]. Our previous study on Seipin^-/-^ mice also revealed increased PA levels in the subventricular zone [65]. Moreover, in yeast, Seipin negatively regulated sphingolipid production by binding with SPT and FA elongase to block LCB and VLCFA production [1]. Our study showed that Seipin deficiency reduced sphingolipid levels (HexCer, Cer, and SM species) due to downregulation its related genes, such as *Cers6, B3gnt2, B4galt4* and *St3gal4*. As sphingolipids were mainly produced by OLs, and its dysmetabolism was implicated in demyelinating disorders [66–68], we proposed that Seipin deficiency down-regulated OPC sphingolipid metabolism, eventually disrupting OPC differentiation and causing abnormal myelination. Liu et al. reported that sphingolipids were accumulated in adipocytes of adipose-specific Seipin knockout mice [69]. To be noted, in mature adipocytes, Seipin promoted fat anabolism and LDs production [69, 70], whereas in adipose precursor cells, Seipin restricted lipogenesis and reduced LDs content [9]. We found that the impacts of Seipin deficiency on LDs content and lipid metabolism were distinct in differentiated and undifferentiated OLN cells. In sum, these findings suggested that Seipin played key roles in modulating lipid metabolism, which was heterogeneous.

Given the pivotal role of Seipin in modulating lipid metabolism, and its deficiency inevitably leads to severe diseases, including the hereditary disorder CGL2. Current treatments for CGL2 are mainly symptomatic, and alternative therapeutic strategies are needed. RG, a PPARγ agonist, was found to ameliorate symptoms of CGL2 patients. RG treatment has shown to promote oligodendrogenesis, white matter repair, and function recovery by promoting oligodendroglia differentiation [71, 72]. RG treatment also alleviate motor or spatial cognitive impairments of Seipin-deficient mice [19], activate genes involved in FA elongation and restore lipid homeostasis [73, 74]. In present study, we reported for the first time that RG treatment promoted OPC differentiation, and myelination, and ultimately alleviated cognitive impairments of Seipin-deficient mice. Mechanistically, we demonstrated that RG activated sphingolipid metabolism-related genes. There were other possible pathways through which RG enhanced OPC differentiation and myelination, including enhanced the expression of alkyl dicarboxylic acid-phosphate tone synthases, stimulated myelin lipid plasmalogen synthesis, reduced oxidative stress, enhanced mitochondrial function, and increased ADP-induced Ca^2+^ wave activity [75–78]. These results supported the therapeutic effects of RG in Seipin deficiency-related diseases.

**Fig. 9:**
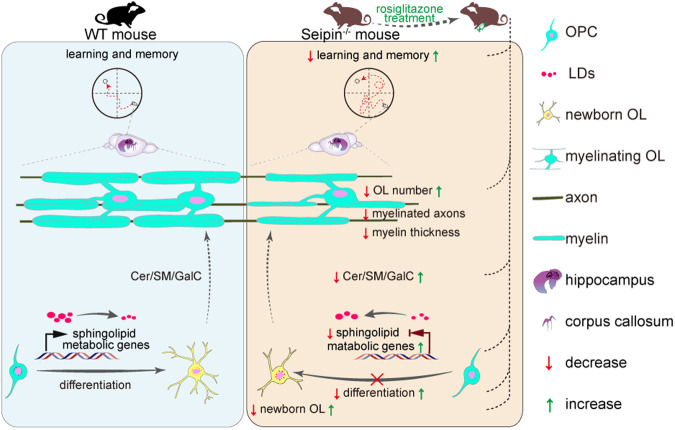
Summary schematic illustrating cognitive deficits induced by Seipin deficiency through impaired OPC differentiation, which is restored by RG administration. Seipin deficiency impaired OPC differentiation, consequent myelination, and spatial cognition. Mechanistically, Seipin deficiency induced lipid dyshomeostasis via interfering expression of sphingolipid metabolism-related genes. RG administration rescued phenotypes induced by Seipin deficiency.

In summary, our study demonstrated that Seipin deficiency compromised OPC differentiation, and consequent myelin abnormalities and neurobehavioral deficits. Mechanistically, Seipin regulated the expression of lipids, especially sphingolipids, metabolism-related genes that underlie OPC differentiation. Meanwhile, RG treatment alleviated the spatial cognition ability of Seipin deficient mice by promoting lipid metabolism (Fig. 9). Present study provides novel insights into the mechanism and paves the ways for the treatment of myelination abnormal-related neurological diseases.

## Materials and methods

### Animals

C57/BL6 mice were obtained from the Animal Center of Shanxi Medical University and Seipin heterozygous-knockout (Seipin^+/-^) lacZ reporter mice were intercrossed to obtain homozygous knockout (Seipin^-/-^) mice. Behavioral tests were conducted during the light phase of the cycle. Adult (3–5-month-old) male mice were used in all experiments. All behavior tests were conducted double-blindly.

### In vivo BrdU corporation

5-bromo-2-deoxyuridine (BrdU; Solarbio, Beijing, Cat# S00135) dissolved in PBS was intraperitoneally injected into adult Seipin^-/-^ and wild type (WT) mice (100 mg/kg) once daily for 7 consecutive days. All mice were sacrificed 14 days after the last BrdU corporation by intracardiac perfusion.

### In vivo RG treatment

RG (MCE, Monmouth Junction, NJ, Cat# BRL 49653) was dissolved in dimethyl sulfoxide at 7.5 mg/mL, and diluted in corn oil to 0.75 mg/mL. Mice received daily intragastric administration of RG (5 mg/kg) or corn oil (as control) for 12 days. Concurrently, to label proliferating cells and track their fate, BrdU (100 mg/kg) was intraperitoneally injected daily for 7 days starting on day 6 of RG treatment. After a 6-day interval, mice underwent Morris water maze (MWM) training and testing. Brains were collected 24 h after the last behavior test for biological analysis.

### Behavior tests

#### MWM test

The MWM (120 cm diameter) testing scheme included a place navigation task and a spatial probe task. During the place navigation task, mice underwent 6 consecutive days of testing, with 4 hidden-platform trials each day and an inter-trial interval of 30 (minutes, min). Released randomly from NE, NW, SW, or SE, mice sought the platform within 1 min; if unsuccessful, they were gently placed on it for 15 seconds. After a 24-h interval, the platform was removed, initiating the spatial probe test. Mice were released farthest from the platform, and swam freely for 1 min. The escape latency, time spent in the target quadrant, and the number of platform location crosses were recorded within a 1-min timeframe.

### Y-maze spontaneous alternation test

A Y-shaped maze with three white, opaque plastic arms at a 120° angle from each other was used. After introduction to the center of the maze, the mice were allowed to freely explore for 8 min. An entry occurred when all four limbs were within the arm. The number of arm entries and the number of triads were recorded to calculate the percentage of alternation.

### Novel object recognition test

A gray acrylic arena (44 × 44 cm) was used. On the day before the test, the mice were allowed to explore an empty arena for 5 min. After 24 h, the mice were allowed to freely explore two identical objects placed at an equal distance for 10 min in the familiar arena. The object exploration and preference were evaluated during these sessions to rule out any eventual intrinsic bias. After 4 h, one of the two objects was replaced with a novel one, then the mice were allowed to explore freely for 10 min. The time spent exploring each object was recorded, and the discrimination index was calculated Discrimination Index = ((Novel Object Exploration Time/Total Exploration Time) × 100) − ((Familiar Object Exploration Time/Total Exploration Time) × 100).

### Accelerating rotarod test

A rotating drum with a diameter of 3 cm was used. Mice were subjected to a 2-day training period, with the first day consisting of a 5-min adaptation trial at 4 rpm, and the second day involving accelerated training from 4 to 40 rpm in 5 min. Then, mice were tested on an accelerating rotarod (4-40 rpm in 5 min). The latency of mice falling off the accelerating rotarod within 5 min was recorded 3 times with a 60-min interval. The average value of 3 latencies was calculated.

### Beam walking test

A 1-m long, 0.2-cm thick, and 0.5-cm wide wooden beam, positioned 50 cm above the ground with a lit starting point, was used. Mice aimed to reach a 20 × 10 × 20 cm^3^ closed box at the end of the beam. Mice were trained for 3 consecutive days to walk across a sequence of 30-, 50- and 70-cm distances to reach the box. On the test day, mice were placed at a 90-cm distance to reach the box. The total time that the animals were on the beam and hindpaw foot slip numbers from the horizontal surface of the beam were recorded. Trials were videotaped for precise timing.

### Mice perfusion and sample collection

Mice were deep anaesthetized with 2.5% isoflurane (RWD Life Science Co., Xingtai, China, Cat# R510-22-10), and transcardially perfused with potassium-free PBS. The brain was isolated immediately, with one hemisphere post-fixed in 4% paraformaldehyde for 24 h for histology, and the other sub-dissected for biochemical analysis. Post-fixed samples underwent 15 and 30% sucrose dehydration (each for 24 h) at 4 °C, and after embedding in OCT (Sakura, Finetek, Japan, Cat# 4583) immediately frozen in liquid nitrogen. Slide-mounted 16 μm or free-floating coronal 30 μm sections were cut using a cryostat (Leica Microsystems, Nussloch, Germany, Cat# CM1860). Brain tissues of the corpus callosum (CC; ~Bregma +0.26 mm to +0.98 mm) and the hippocampus ( ~ Bregma -2.18 mm to -1.46 mm) were collected. Slide-mounted sections were stored at -80 °C, while free-floating sections were stored in cryoprotective medium (30% sucrose/30% ethylene glycol in PBS) at -20 °C.

### X-gal staining and Immunostaining

The Seipin^-/-^ transgene construct included a reporter gene LacZ encoding beta-galactosidase in exon 5-7 of *Bscl2* gene. Beta-galactosidase expression was visualized by X-gal staining using a kit (Beyotime, Shanghai, China, Cat# RG0039). Brain sections were incubated with an X-gal staining solution for 12–18 h at 37 °C. To block endogenous peroxidase activity, sections were incubated in 0.3% H_2_O_2_ containing 70% methanol at room temperature for 30 min. After incubation with 5% normal goat/rabbit serum in 0.3% Triton X-PBS, sections were incubated with the primary antibodies. Following three washes, secondary antibodies were applied at 37 °C for 1 h. After incubation with ABC reagent at 37 °C for 30 min, DAB solution (Proteintech, Wuhan, China, Cat# PR30010) was added until staining developed. Stained sections were then dehydrated and cover-slipped. The primary and secondary antibodies used are listed in Table 1.

**Table 1:** Antibodies used in this study.

Antibodies	Application	Dilution	Company & Catalog number
**primary antibodies**			
Mouse anti-MBP monoclonal IgG	IF or WB	1:200 or 1:1000	Biolegend, Cat# 808401
Rabbit anti-OLIG2 monoclonal IgG	IF or IHC	1:200	Abcam, Cat# ab109186
Goat anti-PDGFRα polyclonal IgG	IF or IHC or ICC	1:200	R&D, Cat# AF1062
Mouse anti-APC (CC1) monoclonal IgG	IHC	1:50	Sigma-Aldrich, Cat# OP80
Mouse anti-BrdU IgG	IF	1:200	Abclonal, Cat# A1482
Rabbit anti-MAG monoclonal IgG	ICC or WB	1:100 or 1:1000	Cell signaling Technology, Cat# 9043
Rabbit anti-Seipin polyclonal IgG	ICC or WB	1:100 or 1:1000	Abcam, Cat# ab106793
Mouse anti-β-actin	WB	1:10000	Abclonal, Cat# AC038
**secondary antibodies**			
Cyanine cy3 donkey anti-mouse IgG	IF	1:200	Jackson, Cat# 715-165-150
Alexa 488 donkey anti-mouse IgG	IF	1:1000	Invitrogen, Cat# A-21202
cy3 donkey anti-rabbit IgG	IF	1:200	Bioss, Cat# bs-0295D
Alexa 488 donkey anti-rabbit IgG	IF	1:1000	Invitrogen, Cat# A-21206
Cyanine cy5 donkey anti-rabbit IgG	IF	1:200	Jackson, Cat# 711-175-152
Alexa 594 donkey anti-goat IgG	IF	1:1000	Invitrogen, Cat# A-11058
Alexa 488 donkey anti-goat IgG	IF	1:200	Jackson, Cat# 705-545-003
Rabbit IgG, HRP	WB	1:5000	ZSGB-BIO, Cat# ZB-2306
Mouse IgG, HRP	WB	1:5000	ZSGB-BIO, Cat# ZB-2305
Biotin Conjugated AffiniPure rabbit Anti-goat IgG (H + L)	IHC	1:100	Boster, Cat# BA1006
Biotin Conjugated AffiniPure goat Anti-rabbit IgG (H + L)	IHC	1:100	Boster, Cat# BA1003
Biotin Conjugated AffiniPure goat Anti-mus IgG (H + L)	IHC	1:100	Boster, Cat# BA1001

### Immunofluorescence

Slide-mounted 16 μm sections were adopted for MBP immunofluorescent staining or Fluoromyelin staining. For BrdU, OLIG2, and PDGFRα triple immunofluorescent staining, free-floating 30 μm coronal sections were selected. The sections were washed in PBS for 5 min, permeabilized with 0.3% Triton X-100 PBS solution for 30 min, washed three times in PBS over 15 min, and blocked in 10% normal donkey serum in PBS with 0.1% Triton X-100 for 1 h at 37 °C. Sections were incubated primary antibodies overnight at 4 °C and were then treated with secondary antibodies at 37 °C for 30 min. Nuclei were counterstained with DAPI (Sigma-Aldrich, Steinheim, Germany, Cat# D9542). For BrdU immunofluorescence, sections were pretreated with 2 N HCL for 16 min followed by 0.1 mol/L boric acid (pH 8.5) for 10 min at 37 °C. Floating sections were mounted onto glass slides, and the fluorescence was preserved by the application of a fluorescent mounting medium (Dako, Hamburg, Germany, Cat# S3023) with the coverslip. The primary antibodies and second antibodies used in this study are listed in Table 1. For myelin lipids staining, the sections were stained with Fluoromyelin Red (1:300; Thermo Fisher Scientific, Paisley, UK, Cat# F34652) at 37 °C for 30 min, and washed with PBS 3 times.

### Cell Culture

OLN-93 cells (termed OLN cells) were cultured in high glucose DMEM (Gibco, Grand Island, USA, Cat# 11965092) supplemented with 10% fetal bovine serum (Gibco, Auckland, New Zealand, Cat# 10099141 C) at 37 °C in 10% CO_2_. After overnight attachment, cells were cultured with serum-free EBSS media and carried on experiments 8 h or 24 h after induction. For depletion of Seipin, OLN cells were transfected with Seipin-short hairpin RNA (sh-Seipin, HANBio, 5’-3’ sense, GCC AAU GUC UCG CUG ACU ATT; 3’-5’ antisense, UAG UCA GCG AGA CAU UGG CTT) for 72 h, or vehicle control short hairpin RNA (NC, 5’-3’ sense, UUC UCC GAA CGU GUC ACG UTT; 3’-5’ antisense, ATG UGA CAC GUU CGG AGA ATT). For LDs induction, OLN cells were supplemented with 0.5 μM Oleic acid (OA; Sigma-Aldrich, Vienna, Austria, Cat# O1383) in 100% ethanol for 16 h. For regulating lipid metabolism, sh-Seipin cells were treated with 1 μM RG for 24 h. For Atglistatin treatment, cells were treated with 25 μM Atglistatin (Sigma-Aldrich, Natick, Massachusetts, US, Cat# SML1075) stock solution for 24 h at the beginning of differentiation.

### Immunocytochemistry

Cells were seeded on poly-d-lysine (Sigma-Aldrich, Vienna, Austria, Cat# P0899)-coated glass coverslips. Fixed cells were permeabilized with 0.1% Triton X-100 in PBS and blocked with normal donkey serum for 1 h. The cells were incubated in primary antibodies overnight at 4 °C and secondary antibody for 1 h at 37 °C. All antibodies used and their dilution factors can be found in Table 1. To observe LDs, boron-dipyrromethene (Bodipy) 493/503 (1 mg/mL; Invitrogen, Eugene Oregon, USA, Cat# D3922) dye staining was performed in 37 °C for 15 min.

### Oil Red O lipid staining

Pre-treated cells were fixed with 4% paraformaldehyde for 15 min, and rinsed with PBS. After a 5-minute incubation in 60% isopropanol and complete drying, cells were stained with Red O (ORO; Sigma-Aldrich, MO, USA, Cat# O1391) working solution for 20 min, followed by three sterile water rinses. Subsequent elution of cellular ORO was performed by gently washing with 100% isopropanol, and the optical density of eluted ORO was measured at 510 nm.

### Fluorescent imaging and quantification

Slices were mounted on slides and imaged by fluorescence microscope (Olympus BX51) and confocal scanning laser microscopy (Olympus FV3000). To remove the background of auto-fluorescence, uniform cutoff thresholds were applied to all captures. A series of images were collected to span either CC or hippocampus, and images covering each region were stitched (NIS-Elements, Nikon) together to produce a single image for analysis with ImageJ/FIJI (NIH, Bethesda, MD, USA), by researchers blinded to the experimental condition. Cell density was calculated by dividing the total number of positive cells (at least 5 sections per mouse) by the total area of these sections.

For assessing the density of OPCs and OLs, counts of individual OLIG2-positive signals were first performed separately, followed by identification and counting of OLIG2 and PDGFRɑ double-positive cells (OPCs) or OLIG2^+^PDGFRɑ^-^ cells (OLs). To assess the differentiation potential of OPCs, all BrdU^+^OLIG2^+^ cells were counted, and the number of OLIG2^+^/BrdU^+^/PDGFRɑ^+^ cells was subtracted from the total BrdU^+^OLIG2^+^ cells number to determine the number of BrdU^+^OLIG2^+^ cells not labeled by PDGFRɑ. All cell counts were normalized to the area quantified to yield cell densities.

The Fluoromyelin or MBP immunofluorescence intensity was measured using the IntDen function of ImageJ/FIJI, and the intensities were further normalized to the average of the control samples of each experiment.

For LDs characterization analysis, including LDs size distributions, number and total LDs area per cell, high-magnification confocal images (100 × objective) of no less than 50 cells from each group were selected from at least 3 independent slides, and subsequent analysis was done with ImagePro-Plus (Media Cybernetics Inc, Bethesda, MD). For quantification of LDs size, z-max intensity projections were segmented using auto-thresholding, followed by watershedding split to separate overlapping LDs. Segmented LDs were analyzed using the particle analysis function (size 0.01-infinity), and ellipses were fitted on each particle to determine the diameter of segmented LDs. A circularity selection of 0.8–1.0 was used to exclude overlapping LDs and aberrant-shaped structures. Size distributions of LDs per cell were based on a previously described protocol (Dollet et al., 2016), by which we have successfully fractionated LDs into large (greater than or equal to 1 μm), medium (between 0.4 μm and 1 μm) and small (small than or equal to 0.4 μm) subpopulations.

For analysis of OLs morphological changes, differentiating OLN cells were categorized into 3 types based on cell morphology: grade 1 (G1) is poorly-differentiated cells, which referred to those extending only one or two primary processes from the cell body; G2 is medium-differentiated cells with multiple processes (three or more); G3 is well-differentiated cells with extensive branches and even develop a complex network.

### Fluorescent in situ hybridization-Immunofluorescence (RNAscope)

Fresh frozen brain sections (16 µm) were stained following the Integrated Co-Detection Workflow combining in situ hybridization using the RNAscope Multiplex Fluorescent v2 assay (ACD bio) and immunofluorescence. After fixation and dehydration, tissues were pretreated with hydrogen peroxide. Sections were washed two times in PBS before incubation with the primary antibodies (OLIG2, PDGFRɑ, or CC1) in co-detection antibody diluent (ACD Bio, Cat# 323160) overnight at 4 °C. Downstream RNAScope processing followed the manufacturer’s instructions. Briefly, sections were treated for 30 min with RNAScope Protease plus, followed by *Bscl2* RNAScope probe (ACD Bio, Cat# 122851) hybridization for 2 h with a HybEZ oven set at 40 °C. Following 3 amplification steps, HRP signals were detected using TSA Plus Cy3 reagents (ACD Bio, Cat# 323272). For immunofluorescence labeling with antibodies following the RNAScope assay, tissues were incubated with Alexa-Fluor-conjugated secondary antibodies in co-detection antibody diluent immediately after developing the HRP-TSA Plus signal. Tissues were counterstained with DAPI. Slides were imaged using an Olympus FV3000 confocal microscope.

### Transmission electron microscopy (TEM)

Seipin^-/-^ and WT mice were anesthetized, perfused with 2% glutaraldehyde/4% paraformaldehyde in 0.1 M phosphate buffer, and post-fixed at 4 °C. Brains parasagittal blocks (1 × 1 × 3 mm) containing the CC (~Bregma +0.5 to -0.5) or hippocampal CA1 (~Bregma -1.0 to -2.0) were dissected, dehydrated in graded ethanol, infiltrated with propylene oxide, and embedded in Epon812 resin. Ultrathin 80 nm sections were cut (Leica Ultra-cut UCT7), stained with uranyl acetate and lead citrate, and imaged (Japan JEM-1011 microscope). Axon diameter, myelin thickness, and the percentage of myelinated fibers were evaluated using methodology published in literature [79]. Specifically, 8-10 random non-overlapping electron micrographs at 12000 × magnification per mouse (3 mice/group) were analyzed. Using the grid tool of ImageJ/FIJI, we unbiasedly assessed axons lying on one or more lines, and characterized them as myelinated or unmyelinated. The proportion of myelinated axons was expressed relative to the total number of fibers assessed. Axon diameters were calculated by measuring the circumference and then calculating the diameter on the basis that the circumferences were circular. Myelin thickness was assessed by measuring the length of the densely myelinated region surrounding the axon. Whole fiber diameter was then calculated as the sum of the axon diameter and twice the myelin thickness. The g-ratio was calculated as axonal diameter/fiber diameter.

### RNA isolation and transcriptome analysis

The total RNA of the brain was isolated by TRIzol reagent (Invitrogen, Carlsbad, CA, USA, Cat# 15596026CN) according to the manufacturer’s instructions. Transcriptome sequencing and data analysis were completed by Biomarker Technologies Company (Beijing, China). To screen the differentially expressed genes (DEGs), raw sequencing data were first cleaned to remove the low-expressed genes and expression data were normalized across samples. The R package DESeq2 (version 1.38.3) was then used to screen for DEGs with *P* < 0.05 and log_2_ FC > 0.5 or <-0.5. For GO enrichment analysis, gene IDs were first mapped using the org.Mm.eg.db package (version 3.16.0), then the clusterProfiler package (version 4.7.1.003) was used to annotate the biological processes (BPs) of the DEGs found, and finally the enrichment results were visualized using the enrichplot package (version 1.18.4).

### Quantitative RT-PCR (qPCR)

Total RNA was extracted from brain samples and cells using RNAiso Plus (Takara, Shiga, Japan, Cat# 9109). Complementary DNA (cDNA) was synthesized using an RT-reagent kit (Takara, Shiga, Japan, Cat# RR047A) followed by the assessment of gene expression using TB Green PCR reagent system (Takara, Shiga, Japan, Cat# RR820A), following the manufacturer’s protocol. Primers were designed with NCBI Primer-Blast or Primer Express 2.0 and synthesized by BGItech (for primer pair sequences, see Table 2).

**Table 2:** Primers used in this study.

Gene name	forward primer	Reverse Primer
**mouse**		
*B4galt4*	AACCCACCTTATCACCTCTCC	TAGCACCCACAAAGTAGTTGC
*Cd36*	ATGGGCTGTGATCGGAACTG	AGCCAGGACTGCACCAATAAC
*Cers6*	GATTCATAGCCAAACCATGTGCC	AATGCTCCGAACATCCCAGTC
*Dgat1*	TCTTAAAGCTGGCGGTCCCC	GACCTGAGCCATCATGGCTG
*Fasn*	GCTGCGGAAACTTCAGGAAAT	AGAGACGTGTCACTCCTGGACTT
*Pgc1α*	TTCATCTGAGTATGGAGTCGCT	GGGGTGAAAACCACTTTTGTAA
*Plpp3*	TCGTCCCTGAGAGTAAGAACG	TGCTTGTCTCGATGATGAGGAA
*Pparγ*	GGAAGACCACTCGCATTCCTT	GTAATCAGCAACCATTGGGTCA
*S1pr2*	ATGGGCGGCTTATACTCAGAG	GCGCAGCACAAGATGATGAT
*Sphk1*	GATGCATGAGGTGGTGAATG	TGCTCGTACCCAGCATAGTG
*St3gal4*	ACCAGCAAATCTCACTGGAAG	CCCTGGAAGCATGGCTCTTTC
*β-actin*	CCTCTATGCCAACACAGTGC	CCTGCTTGCTGATCCACATC
**rat**		
*B4galt4*	ACTTCAACCTCTACACCTGC	CTGTAACGCAACCTGTAGC
*Bscl2*	TTGCCAATGTCTCGCTGACT	AGTGGAGATGATTCGGCCAC
*Cers6*	CCGCCATAACAAAGCATCC	GTTGAATGCTCCGAACATCC
*Mag*	AACTGCACCCTGCTTCTCAG	ACGATGTTGGGGGTGTTGAT
*Pdgfrα*	ACCTTGCACAATAACGGGAG	CAGTTTGATGGACGGGAGTT
*Plin2*	CTCTCGGCAGGATCAAAGAC	CGTAGCCGACGATTCTCTTC
*Plp1*	GGCCGAGGGCTTCTACACCAC	CAGGAGCCCACTGTGGAGCAA
*Plpp3*	CTACAAACACCATCCTAGCG	GACACGAAGAACACTATGCAG
*S1pr2*	AACAGTCACCAAAGTCAGC	TCTGAGTATAAACCGCCCA
*Sphk1*	GAACTACTATGCTGGGCAC	GATTCATGGGTGACAGCTG
*St3gal4*	GCAAATCTCACTGGAAGCTC	TGTCTTCTCGGGAGATGGA
*β-actin*	CTTCCTGGGTATGGAATCCT	TCTTTACGGATGTCAACGTC

### Western blotting

Lysed brain tissue or cells were electrophoresed using sodium dodecyl sulfate-polyacrylamide gel and electro-immunoblotted as described in a paper we previously published [65]. The primary and secondary antibodies were listed in Table 1.

### Matrix-assisted laser desorption ionization time-of-flight mass spectrometry imaging (MALDI-TOF MSI) analysis

The CC or hippocampus of mice was embedded in an M1 medium and snap-frozen in liquid nitrogen. Serial sectioning of frozen tissues with 10 μm thickness was collected by cryostat microtome (Leica Microsystems) and thaw-mounted onto indium tin oxide-coated glass slides. Then, 1 mg/mL O-P, N-C/G matrix was employed to spray the above tissue slices in indium tin oxide with a 0.2 mm nozzle airbrush (NEW-LP, China) and completely dried in a vacuum drier for MALDI imaging analysis. All MALDI-TOF MSI data in the positive-ion mode were obtained with an Ultraflextreme mass spectrometer (Bruker Daltonics, America). The MS calibration was performed with the CHCA matrix, and the m/z range was changed between 500 to 1000. To acquire the higher levels of ionization, the spatial resolution of MALDI MSI was set as 50 μm, and the laser intensity was 70%. The ion images based on lipid molecules were visualized and screened by using SCiLS Lab 2020a software. The structural identification of lipids was conducted by using MetaboScape software (Bruker Daltonica, version 2022b).

### Statistical analysis

All experimental values were expressed as the means ± s.e.m. Data analyses were performed with GraphPad Prism, using statistical tests as indicated in the figure legends. In brief, between-group differences were evaluated using student’s *t*-test, one‐way ANOVA followed by Bonferroni’s post hoc test, or two‐way ANOVA followed by Turkey-Kramer post hoc test. All statistical tests were two-sided, with *P* values < 0.05 considered significant. Experiments were repeated at least 3 times.

### Supplementary information

Supplemental Figures and Figure Legends

Uncropped WB

## Data Availability

All datasets generated and analyzed during this study are included in this published article and its Supplementary Information files. RNA sequencing data from mouse brain have been deposited in SRA under accession code PRJNA974405. Additional data are available from the corresponding author on reasonable request.
