# Supplementary material for: Seipin deficiency-induced lipid dysregulation leads to hypomyelination-associated cognitive deficits via compromising oligodendrocyte precursor cell differentiation
Source: Cell Death Dis. 2024 May 21;15(5):350. doi: 10.1038/s41419-024-06737-z (PMC11109229; doi:10.1038/s41419-024-06737-z)
Supplement: Supplementary file 1 — Supplemental Figures and Figure Legends [file 41419_2024_6737_MOESM1_ESM.pdf]

Supplementary Figure and Figure Legends

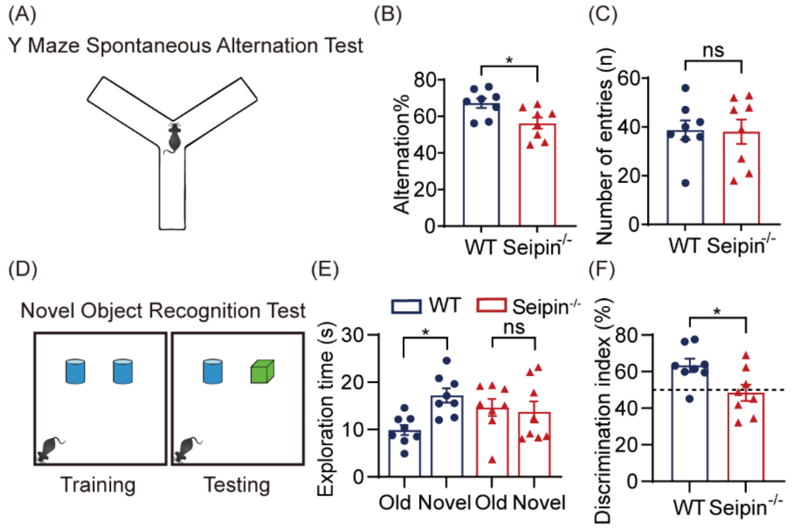

Supplementary Figure 1 Cognitive impairments of Seipin$^{-/-}$ mice validated via Y-maze and novel object recognition assays. **A** Schematic diagram of the Y-maze spontaneous alternation test. **B** Percentage of spontaneous alternation. **C** Total number of arm entries. **D** Schematic diagram of the novel object recognition test. **E** Total exploration time during testing. **F** Discrimination index during testing. Data are expressed as mean ± s.e.m. n=8. ns, not significant; and $^*P < 0.05$. (Unpaired student's *t*-tests).

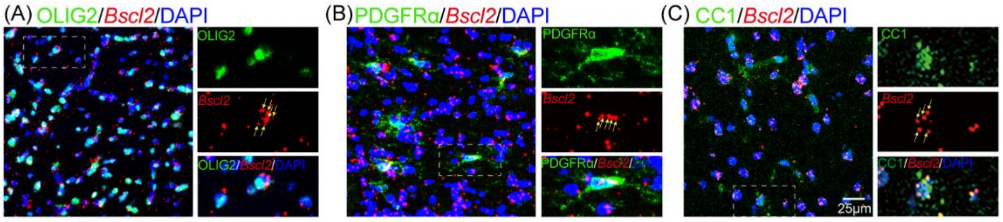

Supplementary Figure 2 Seipin-encoded gene-*Bscl2* was enriched in OPCs and OLs. **A-C** Representative images of *Bscl2* transcripts expression in OL lineage cells (**A**), OPCs (**B**), and OLs (**C**) of 5-month-old WT mice brain. Dashed boxed areas are enlarged. Yellow arrows indicate the *Bscl2* transcript signals.

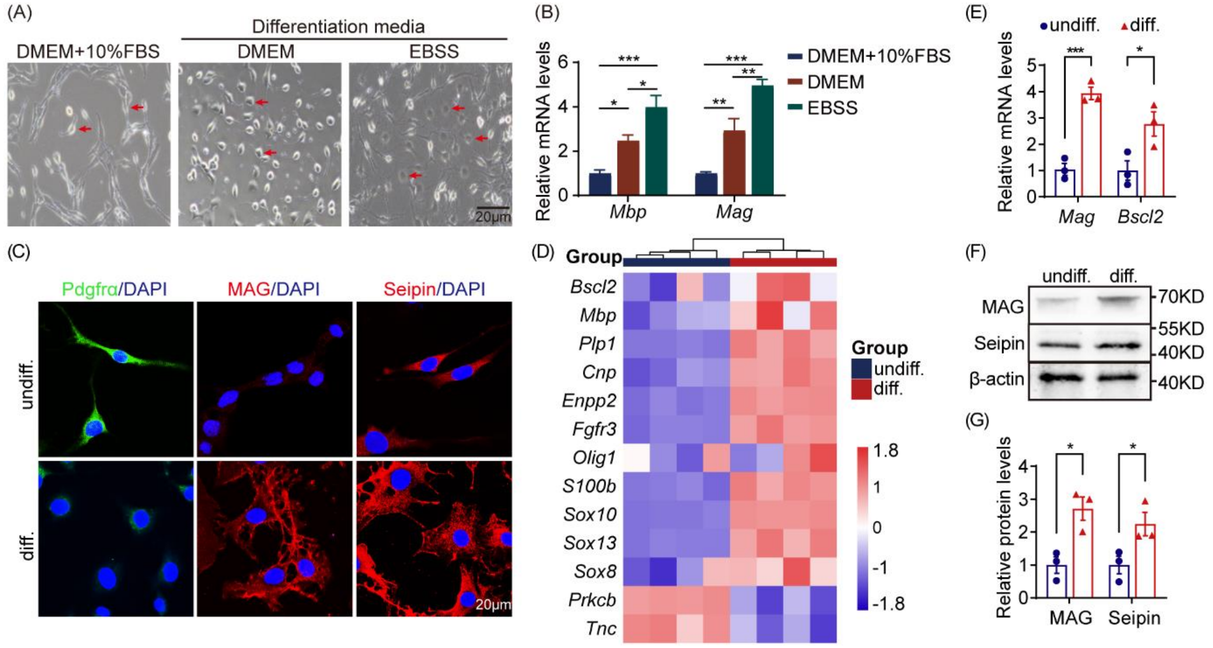

Supplementary Figure 3 EBSS medium promoted OLN cell differentiation better than serum-free medium. A Cell morphology of OLN-93 cells cultured on PDL-coated dishes in three different mediums for 24 h. Undifferentiated OLN cells are bipolar; In DMEM differentiation media, OLN cell bodies extended long bipolar or complex multipolar processes; In EBSS, the cells displayed a more extensive arborization of their processes (red arrows). **B** *Mbp* and *Mag* mRNA levels determined by qPCR in samples of OLN-93 cells cultured in three different media for 24 h. **C** Representative images show PDGFRα (green), MAG (red), and Seipin (red) expression in parallel with morphological changes of the diff- and undiff-OLN cells. **D** Heatmap of differently expressed myelination-associated genes in undifferentiated and differentiated OLN cells from transcriptome-sequencing data. **E-G** Normalized mRNA levels (**E**), immunoblot blots (**F**), and densitometric analysis (**G**) of Seipin and MAG in diff- and undiff-OLN cells. undiff.: undifferentiated. diff.: differentiated for 24 h. Data are expressed as mean ± s.e.m. n=4. **P* < 0.05, ***P* < 0.01 and ****P* < 0.001. (**B** One-way ANOVA followed by Bonferroni's post hoc test; **E and G** Unpaired student’s *t*-tests).

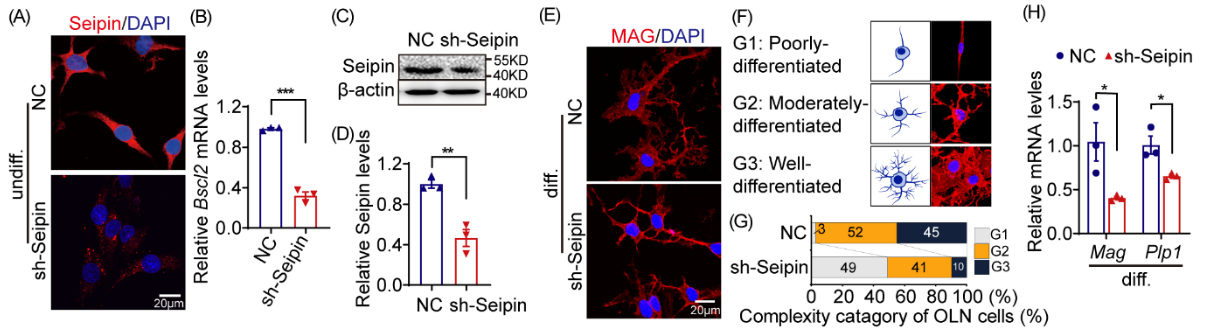

Supplementary Figure 4 Seipin knockdown inhibited OLN cell differentiation. A-D Representative images (**A**), qPCR (**B**), western blotting (**C**) and densitometric analysis (**D**) show relative Seipin expression in OLN cells transfected with NC-shRNA or Seipin-shRNA for 72 h. **E** Representative images show different morphologies of NC and sh-Seipin cells with differentiation for 24 h. **F** Three differentiated categories of OLN cells. **G** Percentage histogram of histological categories of NC and sh-Seipin OLN cells before and after differentiation. **H** The mRNA levels of *Mag* and *Plp1* in differentiated NC and sh-Seipin OLN cells. undiff.: undifferentiated. diff.: differentiated for 24 h. NC: OLN cells transfected with NC-shRNA. Sh-Seipin: OLN cells transfected with Seipin-shRNA. n = 3 triplicate wells per group. **P* < 0.05, ***P* < 0.01, ****P* < 0.001 (**B, D and H** Unpaired student's *t*-tests).

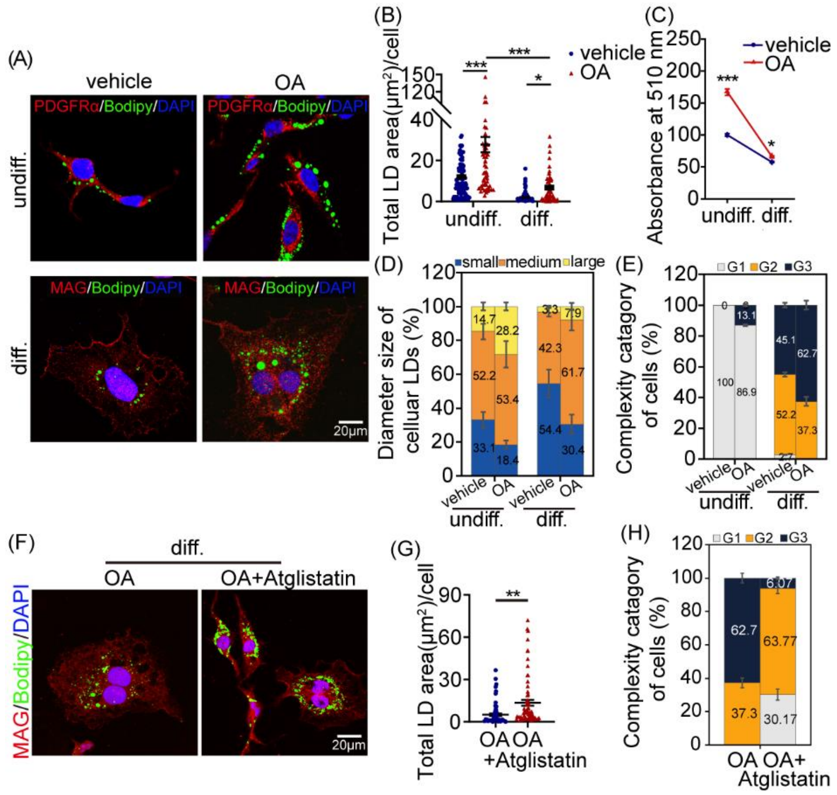

Supplementary Figure 5 Manipulating LDs dynamics altered OLN cell differentiation. **A** Representative images of OA-treated and vehicle-treated OLN cells before or after differentiation for 24 h. **B** Violin plots show LD area per cell in OA-treated and vehicle-treated OLN groups. **C** Quantification of ORO staining. **D** Distribution of different-sized LDs in OA-treated and vehicle-treated OLN cells before and after differentiation. **E** Percentage histogram of histological categories of OA-treated and vehicle-treated OLN cells before and after differentiation. **F** Representative images of differentiated OA and OA+Atglistatin cells. **G** Violin plots show the area of LDs in differentiated OA and OA+Atglistatin cells. **H** Percentage histogram of histological categories of differentiated OA and OA+Atglistatin cells. undiff.: undifferentiated. diff.: differentiated for 24 h. OA: OA-treated cells. OA+Atglistatin: OA-loaded cells treated with Atglistatin. *P < 0.05, **P < 0.01, ***P < 0.001 (Unpaired student's t-tests).

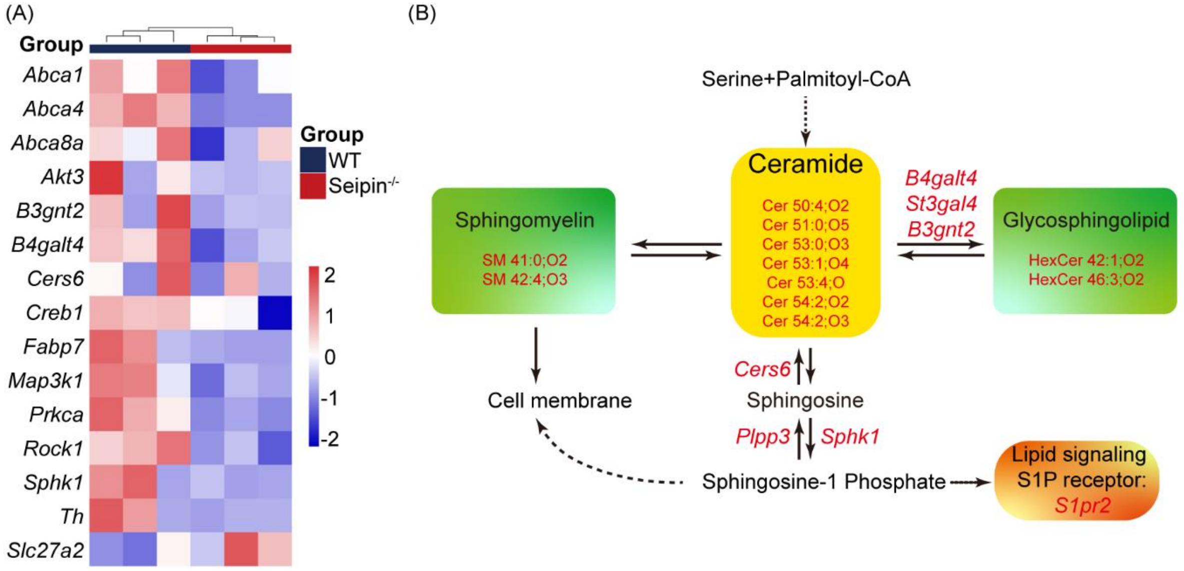

Supplementary Figure 6 Seipin deficiency affected the expression of genes involved in lipid metabolism in brain of mice. A Heatmap of transcriptomic data showing changes in the expression of genes involved in lipid metabolism in brain of Seipin$^{-/-}$ mice compared to WT mice. **B** Schematic representation of the sphingolipid metabolism pathway. Genes and lipid metabolites in red are downregulated by Seipin deficiency.

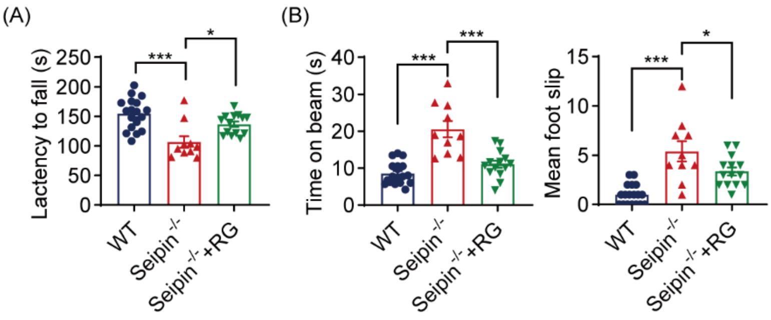

Supplementary Figure 7 RG treatment rescued motor coordination deficits in Seipin$^{-/-}$ mice. A Latency to fall off in the rotarod test. **B** Time to cross the beam and number of foot slips in the beam walking test. Data are expressed as mean ± s.e.m. n=10-18 mice per group. *P < 0.05 and ***P < 0.001. (One-way ANOVA followed by Bonferroni's post hoc test).
