# Supplementary material for: Seipin deficiency-induced lipid dysregulation leads to hypomyelination-associated cognitive deficits via compromising oligodendrocyte precursor cell differentiation
Source: Cell Death Dis. 2024 May 21;15(5):350. doi: 10.1038/s41419-024-06737-z (PMC11109229; doi:10.1038/s41419-024-06737-z)

Full and uncropped western blots for Figure 2C.

Lanes 1, 2, 3, 4, 5, 6 are on the figure.

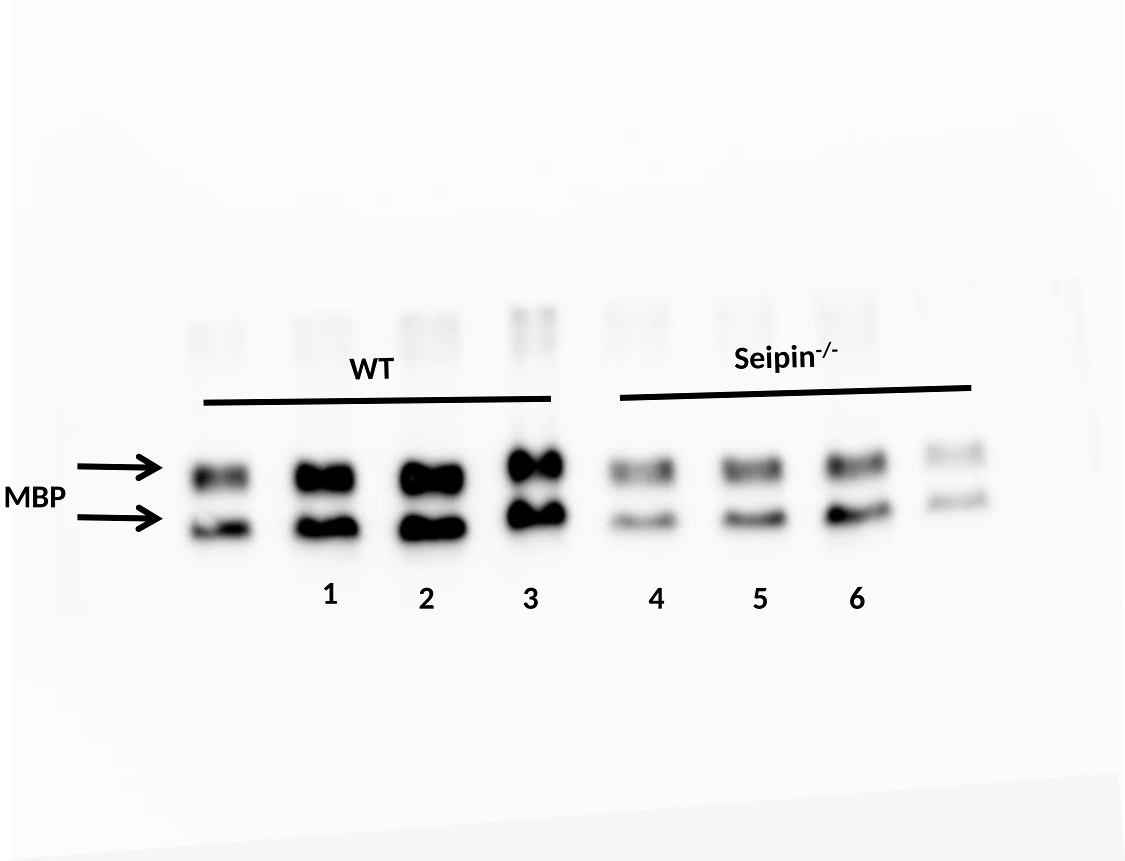

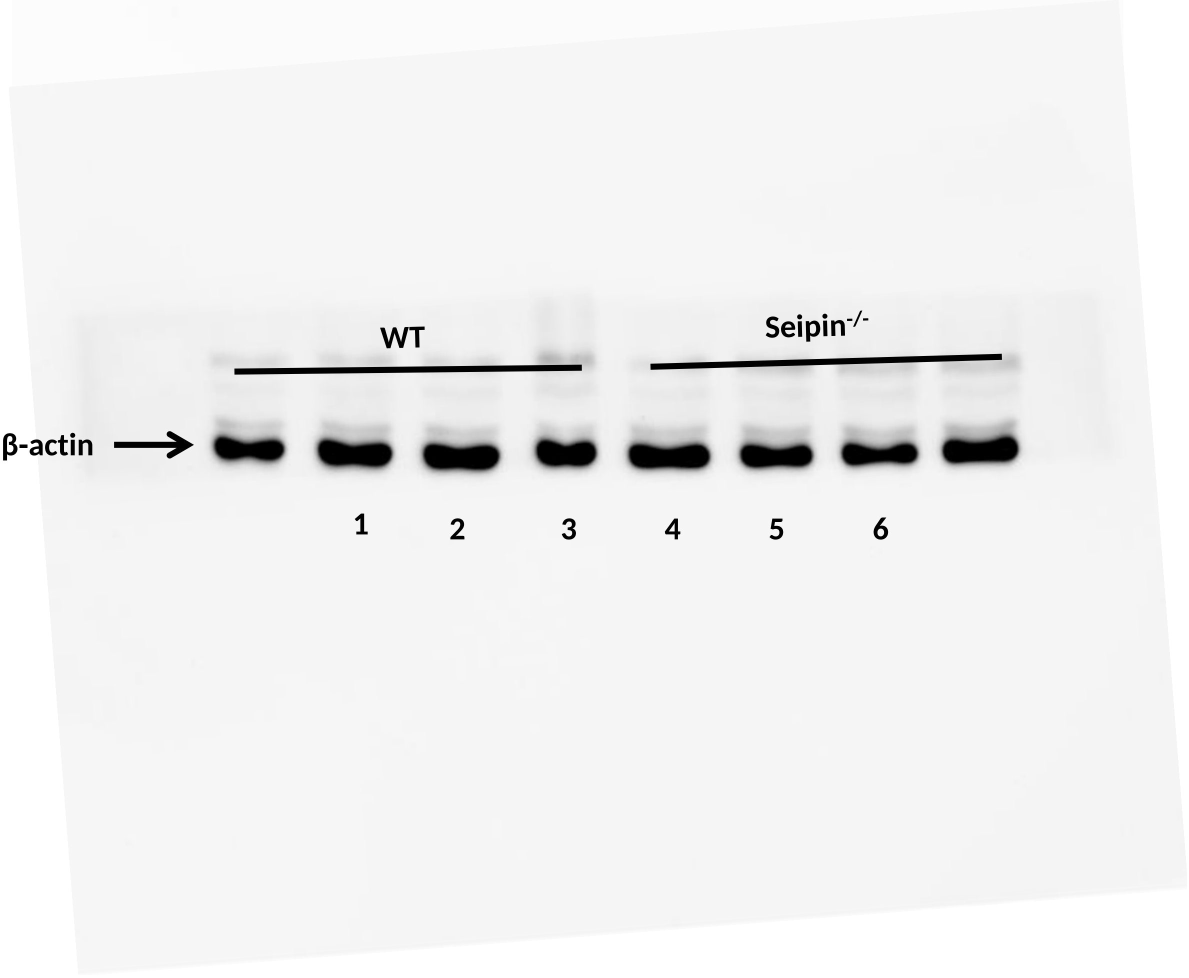

Full and uncropped western blots for Figure 8I.

Lanes 1, 2, 3, 4, 5, 6, 7, 8, 9 are on the figure.

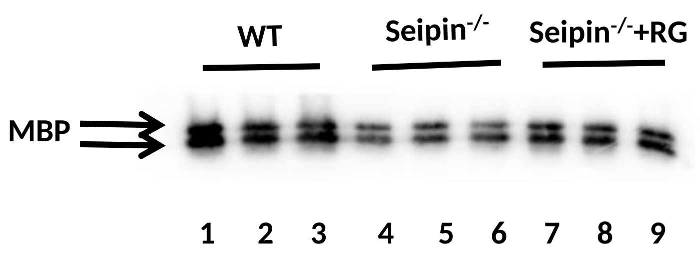

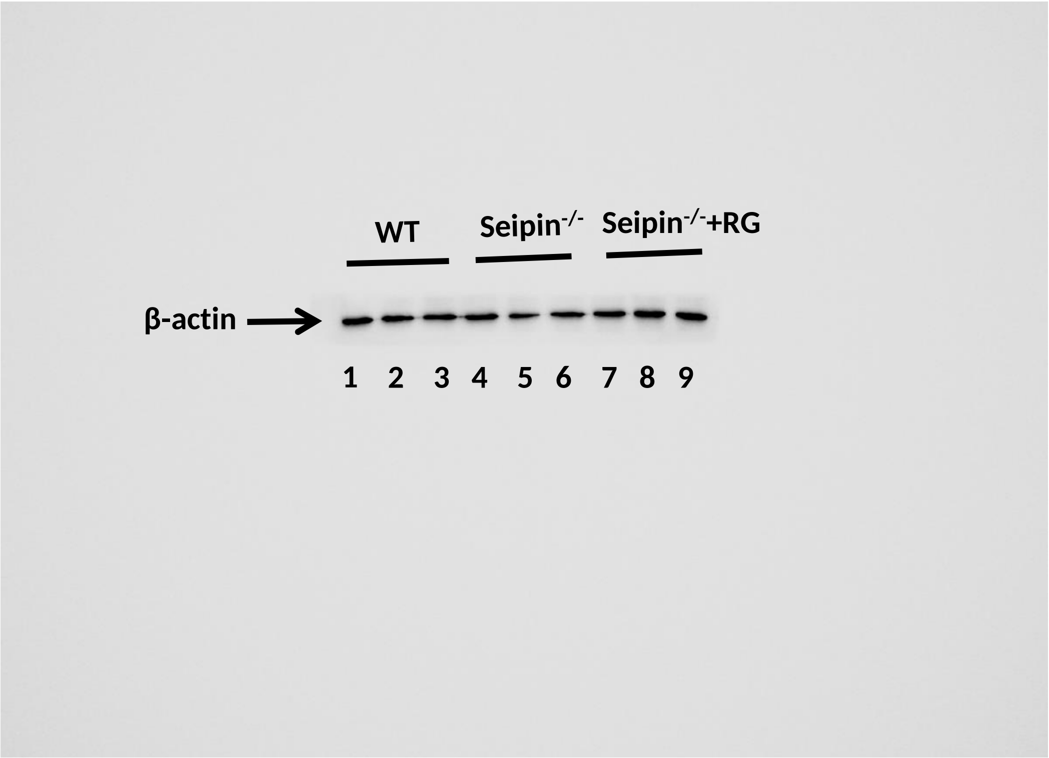

Full and uncropped western blots for Figure S3F.

Lanes 1, 2 are on the figure.

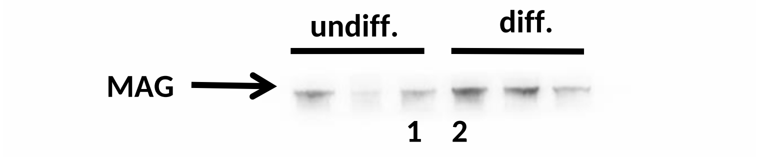

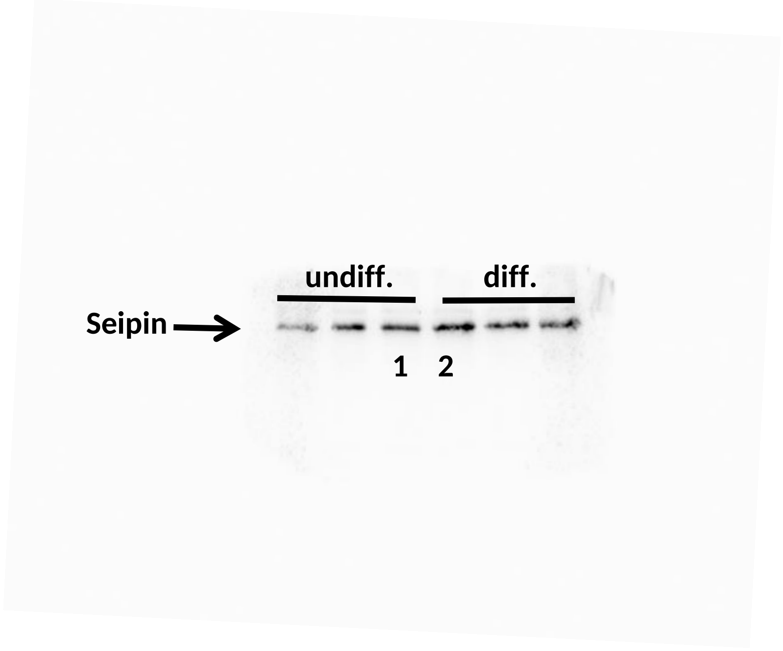

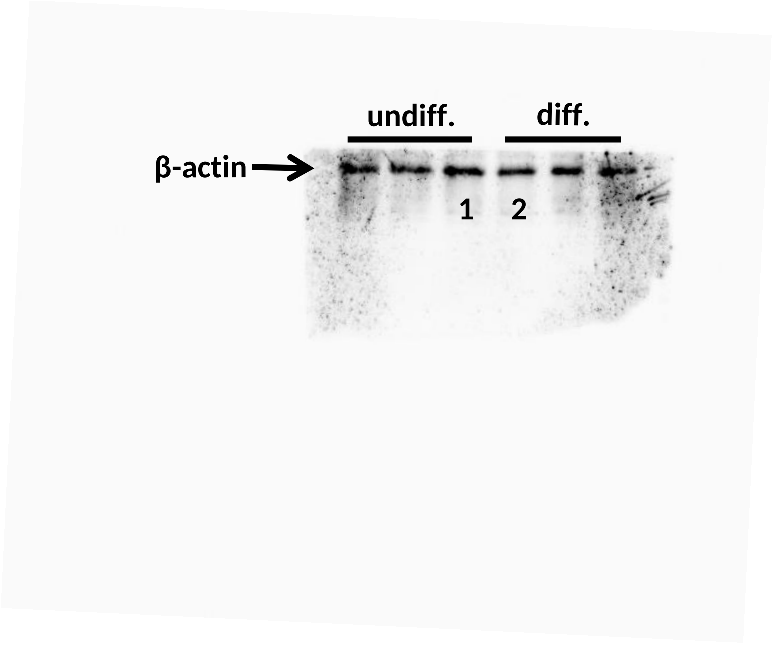

Full and uncropped western blots for Figure S4C.

Lanes 1, 2 are on the figure.

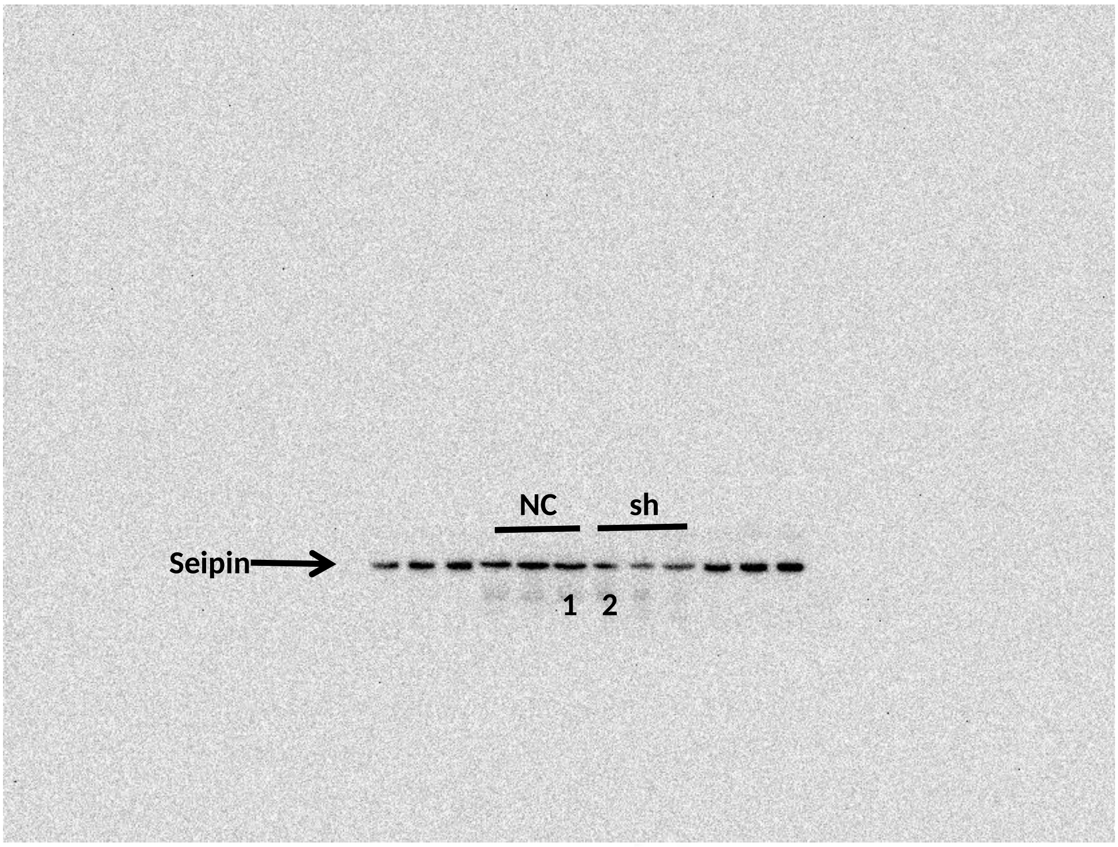

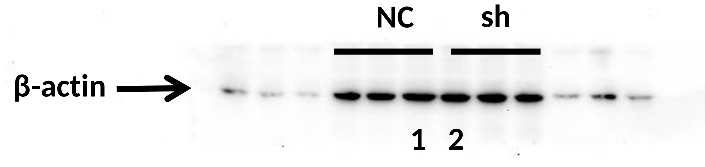

Supplement: Supplementary file 2 — Uncropped WB [file 41419_2024_6737_MOESM2_ESM.pdf]
